# Characterization of the SWI/SNF complex and nucleosome organization in sorghum

**DOI:** 10.3389/fpls.2024.1430467

**Published:** 2024-06-26

**Authors:** Xiaofei Chen, Chao He, Huan Xu, Gongjian Zeng, Quanjun Huang, Zhuying Deng, Xiner Qin, Xiangling Shen, Yongfeng Hu

**Affiliations:** ^1^ Hubei Engineering Research Center for Three Gorges Regional Plant Breeding/Biotechnology Research Center, College of Biological and Pharmaceutical Sciences, China Three Gorges University, Yichang, Hubei, China; ^2^ National Key Laboratory of Crop Genetic Improvement, Hubei Hongshan Laboratory, Huazhong Agricultural University, Wuhan, China

**Keywords:** sorghum, chromatin remodeling, SWI/SNF complex, interaction pattern, nucleosome occupancy

## Abstract

The switch defective/sucrose non-fermentable (SWI/SNF) multisubunit complex plays an important role in the regulation of gene expression by remodeling chromatin structure. Three SWI/SNF complexes have been identified in *Arabidopsis* including BAS, SAS, and MAS. Many subunits of these complexes are involved in controlling plant development and stress response. However, the function of these complexes has hardly been studied in other plant species. In this study, we identified the subunits of the SWI/SNF complex in sorghum and analyzed their evolutionary relationships in six grass species. The grass species conserved all the subunits as in *Arabidopsis*, but gene duplication occurred diversely in different species. Expression pattern analysis in sorghum (*Sorghum bicolor*) showed that most of the subunit-encoding genes were expressed constitutively, although the expression level was different. Transactivation assays revealed that SbAN3, SbGIF3, and SbSWI3B possessed transactivation activity, which suggests that they may interact with the pre-initiation complex (PIC) to activate transcription. We chose 12 subunits in sorghum to investigate their interaction relationship by yeast two-hybrid assay. We found that these subunits displayed distinct interaction patterns compared to their homologs in *Arabidopsis* and rice. This suggests that different SWI/SNF complexes may be formed in sorghum to perform chromatin remodeling functions. Through the integrated analysis of MNase-seq and RNA-seq data, we uncovered a positive relationship between gene expression levels and nucleosome phasing. Furthermore, we found differential global nucleosome enrichments between leaves and roots, as well as in response to PEG treatment, suggesting that dynamics of nucleosome occupancy, which is probably mediated by the SWI/SNF complex, may play important roles in sorghum development and stress response.

## 1 Introduction

Eukaryotic DNA is packed into chromatin, thus preventing the binding of many regulatory proteins for gene transcription. Therefore, dynamics of chromatin structure plays an important role in the transcriptional regulation of genes, which is referred to as epigenetic regulation. The nucleosome, which is the basic unit of chromatin, is subject to both covalent modifications and non-covalent conformational changes, constituting the major mechanism of epigenetic regulation. The conformational changes of chromatin, including nucleosome sliding, histone eviction, and histone replacement, are conducted by Snf2 family chromatin remodeling factors. Phylogenetic analysis reveals that Snf2 family proteins can be divided into at least six groups—Snf2-like, Swr1-like, SSO1653-like, Rad54-like, Rad5/16-like, and distant—among which Snf2-like and Swr1-like groups are the best studied (Flaus et al., 2006). Many chromatin remodeling factors, such as SWI/SNF, ISWI, CHD, SWR1, and INO80, form multisubunit complexes to perform remodeling functions (Clapier et al., 2017).

Although SWI/SNF complexes in different species contain either conserved or specific subunits, the overall 3D structure of these complexes is similar. Taking the yeast SWI/SNF complex as an example, the Arp module is sandwiched between the ATPase module and the body module in the structure (Han et al., 2020). The ATPase domain of the catalytic subunit makes up the ATPase module that binds the nucleosome at superhelical location (SHL) +2. The Arp module is composed of Arp7, Arp9, Rtt102, and the helicase-SANT associated (HSA) domain of Snf2. The conserved subunits Swi1, Swi3, Snf12, and Snf5 and other yeast-specific subunits assemble into the body module. The SWI/SNF complex subunits in *Arabidopsis* have also been identified including the catalytic subunit, BRM/SYD (BRAHMA/SPLAYED), MINU1/2 (MINUSCULE1/2), and other subunits, BSH, SWI3A/B/C/D, SWP73A/B, LFR, TPF1/2, BRD1/2/5/13, BDH1/2, BRIP1/2, GIF1/2/3, and ARP4/7, which correspond to SMARCA-SMARCN excluding SMARCE, SMARCH, and SMARCM (Hernandez-Garcia et al., 2022). However, recently, two groups revealed that there are three SWI/SNF complexes in *Arabidopsis*—BAS, SAS, and MAS—which contain BRM, SYD, and MINU1/MINU2, respectively (Guo et al., 2022; Fu et al., 2023). The composition of the three complexes is divergent, although some subunits are common such as BDH1/2, SWP73A/B, ARP4, and ARP7. In addition, BAS is equivalent to human ncBAF, whereas MAS and SAS evolve several plant-specific subunits such as PSA1/2, SYS1/2/3, and SHH2. The three complexes have both overlapping and specific functions to regulate chromatin accessibility (Guo et al., 2022; Fu et al., 2023).

Most of the SWI/SNF complex subunits have been revealed to play important roles in multiple processes of plant development, such as leaf, root, floral organ and seed development, flowering time control, shoot apical meristem maintenance, chlorophyll biosynthesis, and response to phytohormone (Shang and He, 2022). The multi-copies of some subunits exhibit redundant functions like MINU1/2, BRD1/2/13, BRIP1/2, BDH1/2, and TPF1/2 (Sang et al., 2012; Yu et al., 2020; Jaronczyk et al., 2021; Diego-Martin et al., 2022; Stachula et al., 2023). On the contrary, SWI3 proteins act non-redundantly to control different aspects of development (Sarnowski et al., 2005). Likewise, SWP73B functions more important than SWP73A, as mutation of *SWP73B* severely affects plant development, while *swp73a* mutants only display early flowering (Sacharowski et al., 2015). Additionally, SWP73B is the common subunit shared by three SWI/SNF complexes, while SWP73A is only incorporated into the BAS complex (Guo et al., 2022). The SWI/SNF complex is also involved in regulating environmental stress responses such as salt, drought, and high-temperature stresses, DNA damage, and pathogen attacks (Song et al., 2021). This indicates that the SWI/SNF complexes are the common regulators that target far-ranging loci for remodeling chromatin structure.

In maize, the TAP experiment was performed using AN3 (GIF1) as the bait, which revealed a part of the conserved subunits of the SWI/SNF complex (Nelissen et al., 2015). The protein–protein interactions between some SWI/SNF subunits were also proved in rice (Qi et al., 2020; Guo et al., 2022). However, whether the three SWI/SNF complexes are present in grass remains to be disclosed. In this study, we used protein sequences of the SWI/SNF complex in *Arabidopsis* to search for their homologs in six grass species. The results showed that the grass genomes encoded all the subunits of the SWI/SNF complex, although the homology of some subunits is very low. Expression pattern analysis indicates that the SWI/SNF2 complex genes were expressed in most of the tissues in sorghum at different levels. We revealed the transactivation activity of SbAN3, SbGIF3, and SbSWI3B by the detection in yeast, which suggests that they may be involved in the association with pre-initiation complex (PIC) to promote transcription. Multiple protein–protein interaction analysis by yeast two-hybrid (Y2H) unraveled that the interaction between SWI/SNF subunits in sorghum is very different from that in *Arabidopsis*, implying that novel SWI/SNF complexes probably exist in sorghum. Nucleosome organization features and dynamics between roots and leaves, as well as in response to PEG treatment, were also characterized by MNase-seq and RNA-seq analyses. Our study lays the foundation for further investigating the function of the SWI/SNF complex in the development or stress response of sorghum.

## 2 Materials and methods

### 2.1 Phylogenetic analysis

*Arabidopsis* SWI/SNF complex protein sequences were collected from the Phytozome database (http://www.phytozome.net/poplar) to perform BLAST and obtain the homologous protein sequences in six grass species, including *Sorghum bicolor*, *Oryza sativa*, *Zea mays*, *Hordeum vulgare*, *Brachypodium distachyon*, and *Setaria italica*. The conserved domains of these proteins were analyzed in the PFAM database (http://pfam-legacy.xfam.org/) and confirmed in the SMART database (http://smart.embl-heidelberg.de/).

Multiple sequence alignment of protein sequences was conducted using the software DNAMAN. The unrooted phylogenetic trees of SWI/SNF complex proteins were constructed by MEGA 5 using the maximum likelihood (ML) method with the following parameters: multiple alignment gap opening penalty to 3, the multiple alignment gap extension penalty to 1.8, and a bootstrap test of 1,000 replications. The phylogenetic trees were visualized using ITOL (https://itol.embl.de/upload.cgi).

### 2.2 Analysis of gene structure and chromosomal location

The gene structure display server (GSDS) program (http://gsds.gao-lab.org/) was used to display exon/intron organization for each SWI/SNF complex gene in sorghum by comparing cDNA sequences with their corresponding genomic DNA sequences. Chromosomal locations of sorghum SWI/SNF complex genes were visualized using TBtools software.

### 2.3 Prediction of protein–protein interactions

Search Tool for the Retrieval of Interaction Gene/Proteins (STRING) (https://cn.string-db.org/) was used to predict interactions between SWI/SNF complex subunits based on both direct physical interactions and indirect functional dependency. The diagram was modified using Cytoscape software.

### 2.4 Collection of expression data and promoter *cis*-acting element prediction

RNA-seq expression data were generated by Davidson et al. (2012); Makita et al. (2015), and Wang et al. (2018), and Fragment Per Kilobase of transcript per Million mapped reads (FPKM) values were downloaded from Plant Expression ATLAS (https://www.ebi.ac.uk/gxa/plant/experiments) database. Visualization of expression data and cluster analysis of expression patterns were performed using the TBtools software.

The promoter sequences of SWI/SNF complex genes were extracted from the Phytozome database and were submitted to the Plant Cis-Acting Regulatory Element (CARE) database (http://bioinformatics.psb.ugent.be/webtools/plantcare/html/) for prediction of *cis*-acting elements. The number of each *cis*-acting element was visualized using TBtools software.

### 2.5 Vector construction and yeast two-hybrid assay

Two-week-old seedlings of the BTx623 sorghum variety were used in this experiment for RNA extraction with TRIzol reagents (Invitrogen, Carlsbad, CA, USA). The total RNAs were reverse-transcribed to cDNA with SuperScript® IV Reverse Transcriptase (Invitrogen). The cDNAs of 12 SWI/SNF complex genes were amplified using specific primers, which are listed in **Supplementary Table 1**, and then inserted into the pGADT7 (AD) and pGBKT7 (BD) vectors by ClonExpress® II One Step Cloning Kit (Vazyme, Nanjing, China). The recombinant or empty vectors (AD and BD) were co-transformed into yeast strain AH109, which were then grown for 3–5 days at 30°C on SD/-Trp-Leu plates. Positive clones were dotted on SD/-Trp-Leu and SD/-Trp-Leu-His agar plates with 5 mg/mL of X-α-gal (ZOMANBIO, Beijing, China) for screening.

### 2.6 Plant growth conditions and PEG treatment

Sorghum (BTx623 variety) seeds were surface-sterilized and then soaked for germination. The germinated seeds were transferred to the nursery box (1/2 MS) to continue growing in a growth room kept at 28°C with a 12 h light/dark cycle. The 14-day-old seedlings were transferred to the nursery box containing 20% PEG6000 that was prepared with 1/2 MS. The seedlings transferred to fresh 1/2 MS solution were used as the control. After 6 hours of treatment, the third leaves and roots were harvested for subsequent experiments.

### 2.7 RNA-seq

The sorghum young leaves were harvested and frozen immediately in liquid nitrogen. Total RNA was extracted using TRNzol Universal Reagent (TIANGEN, Beijing, China) following the manufacturer’s instructions. The quality of RNA was measured by gel electrophoresis, NanoDrop analyzer, LabChip, and Qubit analyzer. mRNA was purified using oligo(dT) and then fragmented by incubating in a fragmentation buffer. The fragmented mRNA was primed with random hexamer primers and reverse-transcribed with Reverse Transcriptase. After end repair, adenylation, adaptor ligation, purification, PCR amplification, and quality control, sequencing was performed on an Illumina HiSeq system.

### 2.8 MNase-seq

The samples were crosslinked with 1% (v/v) formaldehyde. The nuclei were extracted using extraction buffer I [0.4 M sucrose, 10 mM Tris HCl pH = 8.0, 10 mM MgCl_2_, 5 mM β-mercaptoethanol, 0.1 mM phenylmethylsulfonyl fluoride (PMSF), and protease inhibitor], extraction buffer II (0.25 M sucrose, 10 mM Tris HCl pH = 8.0, 10 mM MgCl_2_, 1% Triton X-100, 5 mM β-mercaptoethanol, 0.1 mM PMSF, and protease inhibitor), and extraction buffer III (1.7 M sucrose, 10 mM Tris HCl pH = 8.0, 2 mM MgCl_2_, 0.15% Triton X-100, 5 mM β-mercaptoethanol, 0.1 mM PMSF, and protease inhibitor). The nuclei were resuspended with Micrococcal Nuclease (MNase) buffer, and then bovine serum albumin (BSA) and MNase (M0247, NEB, Ipswich, MA, USA) were added and then digested at 37°C for 5 min, 10 min, and 15 min. The reaction was stopped by adding EGTA. RNase and 20 μL 5M NaCl were used for removing RNA and de-crosslinking, respectively, by incubating at 65°C overnight. DNA was purified and separated by 2% agarose gel electrophoresis. Approximately 200 bp of DNA fragments generated by digestion at a proper time was recovered using a gel extraction kit. The libraries were constructed and then sequenced on an Illumina HiSeq system.

### 2.9 Raw sequencing read filtering

To obtain high-quality clean reads of RNA-seq and MNase-seq, the raw sequencing reads were trimmed with Trimmomatic (version 0.32) (Bolger et al., 2014). The Trueseq3-PE adapters were removed using a maximum of two seed mismatches, a palindrome clip threshold of 30, and a simple clip threshold of 10. The leading and tailing bases with quality below 20 or N bases were cut. Read lengths shorter than 36 bp or with average quality per base in the 4-base wide sliding window below 15 were discarded.

### 2.10 Analyses of RNA-seq and MNase-seq data

Transcript abundance was quantified directly using pseudoalignment of high-quality clean RNA-seq reads to the reference cDNA sequences and gene models from Sorghum_bicolor_NCBIv3 assembly of the variety BTx623 (McCormick et al., 2018), as implemented in Kallisto (version 0.48.0) (Bray et al., 2016). The transcripts per million (TPM) and gene count matrices were created using tximport (version 1.22.0) in R (Soneson et al., 2015).

The MNase-seq high-quality clean reads were aligned against the reference Sorghum_bicolor_NCBIv3 genome assembly using Bowtie2 (version 2.4.4) (Langmead and Salzberg, 2012) with the parameters “bowtie2 –no-unal –threads 16 –sensitive -k 3 -q –phred33 –rg-id ‘“$i”_R1_”$i”_R2’ –rg ‘SM:”$i”_R1_”$i”_R2\tPL: Illumina\tLB: Illumina_1_8’”. Aligned reads with Mapping Qualities (MAPQ) < 5 were filtered using SAMtools (version 1.9) (Li et al., 2009) with parameters “samtools view -F 1804 -q 5”. Duplicated alignments were removed using Picard (version 2.23.9) (http://broadinstitute.github.io/picard/) with parameters “picard MarkDuplicates REMOVE_DUPLICATES=true”. Two replicate bam files were merged and converted to a bed file using the “bamtobed” command of BEDTools. The genome-wide nucleosome positions were detected using iNPS (version 1.2.2) (Chen et al., 2014) with the command line “python3 iNPS_V1.2.2.py -i INPUT.bed -o OUTPUT –s_p p”. To draw the distribution curves of nucleosomes, the upstream and downstream 1 kb regions from transcription start site (TSS) were divided into 20-bp bins using “computeMatrix” tool of deepTools with the parameters “computeMatrix reference-point –referencePoint TSS -b 1000 -a 1000 –binSize 20 –skipZeros –averageTypeBins mean “. Then, the “plotProfile” tool of deepTools was used to visualize the distribution of nucleosomes.

## 3 Results and discussion

### 3.1 Identification of SWI/SNF complex in grass

**Figure 1:**
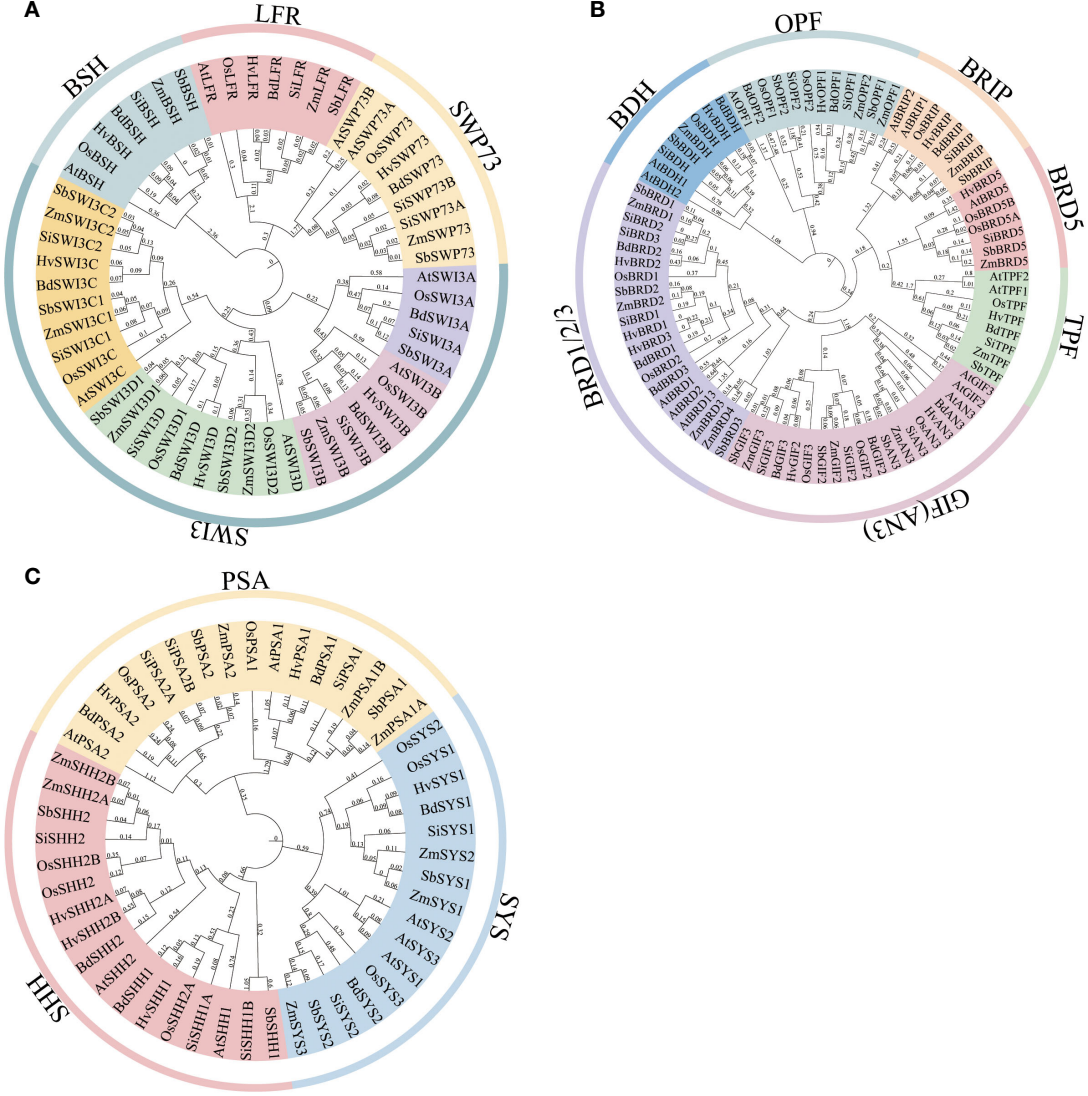
Phylogenetic trees of the SWI/SNF complex subunits in six grass species. **(A)** The core subunits of the SWI/SNF complex include BSH, SWP73, SWI3, and LFR. **(B)** The other subunits of the SWI/SNF complex include BDH, BRD, BRIP, GIF, TPF, and OPF. **(C)** The plant-specific subunits of the SWI/SNF complex include PSA, SHH2, and SYS. Full-length protein sequences of the SWI/SNF complex subunits from *Arabidopsis* and six grass species were used for constructing phylogenetic trees. The locus information of the subunits is provided in **Supplementary Table 2**.

To investigate whether the composition of the SWI/SNF complex is conserved among grass species, the protein sequences of SWI/SNF complex subunits in *Arabidopsis* were used for searching their homologs in sorghum, rice, maize, barley, *B. distachyon*, and *S. italica*. The results indicate that all subunits identified in *Arabidopsis* also exist in six grass species, although gene duplication events for some subunits occur differentially in different species (**Table 1**; **Supplementary Table 2**). The core subunits of the SWI/SNF complex in *Arabidopsis* are BSH, SWI3A/B/C/D, and SWP73A/B, which may constitute the body module of the complex as their counterparts in animals and yeast (Mashtalir et al., 2018; Han et al., 2020). BSH is the homolog of human SMARCB, which is characterized to bind the nucleosome acidic patch by its C-terminal domain, and the binding is important for the remodeling activity and DNA accessibility of the complex (Valencia et al., 2019). All grass BSH proteins are coded by single-copy genes and contain a SNF5 domain (**Figure 1A**; **Supplementary Figure 1**). In addition, sequence alignment showed that the C-terminus of plant BSH proteins also contain several basic amino acids that are responsible for the binding of nucleosome acidic patch in human SMARCB, although the sequence similarity between BSH and SMARCB is very low (**Supplementary Figure 2**), suggesting possible conserved nucleosome-binding activity of these proteins. Two SMARCC subunits serve as the scaffold to bridge all the other core subunits in the human pBAF complex (Mashtalir et al., 2018). Their homologs in *Arabidopsis* are coded by four genes: AtSWI3A, AtSWI3B, AtSWI3C, and AtSWI3D. The four AtSWI3 proteins exhibit non-redundant regulatory and developmental functions possibly by assembling different SWI/SNF complexes (Sarnowski et al., 2005). Phylogenetic analysis showed that divergent evolutionary events occurred for SWI3 genes in different grass species (**Figure 1A**). For example, maize and barley genomes lost SWI3A homologs. SWI3C genes duplicated in maize, sorghum, and *S. italica*; SWI3D genes duplicated in maize, sorghum, and rice. In addition, the gene duplication events of each of SWI3C and SWI3D occurred before the divergence of these species. All six grass species possess a single copy of SWI3B. SWI3 proteins all have the SWIRM domain and the SANT domain that are required for the interaction with the other subunits (**Supplementary Figure 1**). The ZnF_ZZ domain is less conserved in SWIA, SWIB, and part of SWIC. Two *SWP73* genes (*SWP73A* and *SWP73B*) in *Arabidopsis* display distinct functions (Sacharowski et al., 2015). Mutation of *SWP73B* results in multiple severe defects in vegetative and reproductive development, while *SWP73A* only affects flowering time. This suggests the leading role of SWP73B in the composition of the SWI/SNF complex. We found that among six grass species, SWP73 was encoded by two genes only in *S. italica*, while in the other species, it was encoded by one gene (**Figure 1A**). SWP73 proteins contain the conserved SWIB domain involved in the association with the other subunits of the complex (**Supplementary Figure 1**). ARID1A/B, which acts to stabilize the body module, is the largest subunit in the human BAF complex (Mashtalir et al., 2018). Their homologs are absent in plant genomes. However, recently, it was considered that LFR could be the substitute for ARID1A/B in plant SWI/SNF complex (Hernandez-Garcia et al., 2022), as it contains the truncated BAF250_C (or ARM-repeat) domain (**Supplementary Figure 1**). However, LFR lacks the ARID domain and is much smaller than ARID1A/B. Thus, whether LFR can functionally replace ARID1A/B remains to be proved. Surprisingly, we found that plant LFR proteins exhibited extremely high sequence similarity, especially among grass species (**Supplementary Figure 3**).

**Table 1:** The SWI/SNF complex subunits in sorghum.

Subunit	Gene ID	Amino acid (AA)	Molecular weight	pI	Hydrophilic	Subcellular localization
SbBSH	Sobic.009G036200	255	29,076.93	5.87	−0.618	Nucleus
SbLFR	Sobic.004G198900	458	50,024.05	6.05	−0.215	Chloroplast
SbSWI3A	Sobic.006G121300	556	61,121.04	6.47	−0.523	Nucleus
SbSWI3B	Sobic.004G077600	499	54,582.37	5.66	−0.464	Nucleus
SbSWI3C1	Sobic.005G064000	777	84,048.32	6.11	−0.457	Nucleus
SbSWI3C2	Sobic.008G057000	775	83,234.03	6.57	−0.325	Nucleus
SbSWI3D1	Sobic.001G109800	910	98,860.97	5.01	−0.676	Nucleus
SbSWI3D2	Sobic.006G008300	904	98,508.22	4.99	−0.622	Nucleus
SbBRD1	Sobic.001G518100	642	69,505.22	9.26	−0.96	Nucleus
SbBRD2	Sobic.002G288000	585	64,156.88	9.32	−0.893	Nucleus
SbBRD3	Sobic.002G315200	1248	137,306.9	9.11	−0.911	Nucleus
SbSWP73	Sobic.002G394800	533	58,117.56	9.66	−0.485	Nucleus
SbBRIP	Sobic.008G093200	357	39,822.85	4.91	−0.93	Nucleus
SbAN3	Sobic.001G101700	226	23,469.17	5.4	−0.489	Nucleus
SbARP7	Sobic.001G234200	361	39,416.97	4.68	−0.046	Cytoplasm
SbTPF	Sobic.008G113100	818	89,932.81	8.66	−0.813	Nucleus
SbBDH	Sobic.007G102800	215	22,730.42	4.9	−1.139	Nucleus
SbARP4	Sobic.001G536000	444	48,709.18	5.16	−0.31	Cytoplasm
SbGIF2	Sobic.005G187500	215	22,753.68	4.96	−0.59	Nucleus
SbGIF3	Sobic.008G100700	187	19,919.48	4.81	−0.597	Nucleus
SbOPF1	Sobic.004G258700	782	81,996.02	6.57	−0.459	Nucleus
SbOPF2	Sobic.010G130100	679	73,938.99	8.28	−0.565	Nucleus
SbPSA1	Sobic.007G027700	385	42,012.7	5.84	−0.669	Nucleus
SbPSA2	Sobic.003G000800	290	30,724.33	6.96	−0.544	Cytoplasm, Nucleus
SbSHH1	Sobic.005G082300	291	33,235.63	8.75	−0.712	Nucleus
SbSHH2	Sobic.002G165800	398	44,024.43	6.62	−0.603	Chloroplast, Nucleus
SbBRD5	Sobic.007G009400	615	67,575.33	5.16	−1.061	Nucleus
SbSYS1	Sobic.001G457900	1556	169,813.6	6.55	−0.832	Nucleus
SbSYS2	Sobic.001G085000	914	99,579.57	4.97	−0.46	Nucleus

The other accessory subunits in *Arabidopsis* include GIF1/2/3, BRIP1/2, ARP4/7, BRD1/2/13, BRD5, OPF1/2, TPF1/2, BDH1/2, PSA1/2, SYS1/2/3, and SHH2. GIF1, GIF2, and GIF3 contain the SSXT domain and are analogous to SS18 in the BAP complex (**Supplementary Figure 1**). BRIP1/2 possessing the GLTSCR1 domain is the homolog of GLTSCR1 (**Supplementary Figure 1**). Bromodomain-containing BRD1/2/13 and BRD5 are homologous to BRD7 and BRD9. Recently, IP-MS and yeast two-hybrid experiments revealed that TPF1/2 were the subunits of *Arabidopsis* SWI/SNF complex and directly interacted with LFR and SWP73B by PHD domains (Diego-Martin et al., 2022), which is similar to their distant ortholog DPF2 in BAF complex. In the same experiments, BDH1 and BDH2 were also identified in the TPF1-purified complex and considered BCL7A/B/C orthologs, although direct evidence is lacking. Interestingly, different from *Arabidopsis*, we found that BRIP, TPF, and BDH in six grass species were all encoded by a single gene by homologous search (**Figure 1B**), indicating that gene duplication did not occur in these species. Sequence alignment of BDH proteins showed that a conserved region in the N-terminus was present in *Arabidopsis* and grass species (except barley) (**Supplementary Figure 4**), although no domain was predicted in these proteins by SMART (**Supplementary Figure 1**). Other than the conserved N-terminal region, the remaining part of the proteins displayed low similarity between *Arabidopsis* and grass, suggesting that the conserved region may be important for the function of BDH proteins. By contrast, similar to *Arabidopsis*, *OPF* and *GIF* genes were duplicated to give rise to two *OPF*s and three *GIF*s in six grass species (**Figure 1B**). *OPF* genes are also considered the homologs of human *DPF1*/*2*/*3* in the plant due to the existence of the PHD domain in OPF proteins (Hernandez-Garcia et al., 2022). In our opinion, *AN3* (*GIF1*) could be the ancestral gene of the other *GIF*s, as its orthologs in eudicot (*Arabidopsis*) and monocots (grass) reside in the same clade of the tree, while those of *GIF2* and *GIF3* in these species were clustered in the other clade (**Figure 1B**). The number of BRD genes varies in different species, which is four in rice, sorghum, *S. italica*, *B. distachyon*, and barley and five in maize (**Figure 1B**). To identify ARP proteins, we collected all the actin domain-containing proteins and performed phylogenetic analysis. The actin proteins and ARP proteins were clearly separated in the phylogenetic tree (**Supplementary Figure 5**). ARP proteins were clustered into eight classes, which were named ARP2–9 based on their homology with *Arabidopsis* counterparts. All ARP7 proteins and most ARP4 proteins (except in barley) in six grass species are encoded by one gene.

The plant-specific subunits of the SWI/SNF complex include PSA1/2, SYS1/2/3, and SHH2. PSA1 and PSA2 are mostly encoded by one gene in six grass species (**Figure 1C**). However, PSA1 in maize and PSA2 in *S. italica* are encoded by two genes. All six grass species but not *Arabidopsis* PSA2 proteins have the conserved RWP-RK domain (**Supplementary Figure 1**). Consistently, sequence alignment showed low similarity of PSA1 and PSA2 proteins between *Arabidopsis* and grass species (**Supplementary Figures 6**, **7**). Indeed, we found that PSA2 in rice was also named OsRKD1, which belongs to the RKD family transcription factor. However, phylogenetic analysis indicates that OsRKD1 resides in different subfamilies from all *Arabidopsis* RKDs (Chardin et al., 2014), which suggests that OsRKD1 and its grass homologs may evolve novel function in the SWI/SNF complex. *SYS* genes are duplicated in most of the six grass species except barley and are divided into two clades in the phylogenetic tree (**Figure 1C**). We could not predict the conserved domain in SYS proteins in the PFAM database (**Supplementary Figure 1**), but we identified a segment of conserved sequence at the C-terminal of SYS proteins by sequence alignment (**Supplementary Figure 8**). SHH2 is also a transcription factor that contains the HOX domain and the SAWADEE domain (**Supplementary Figure 1**) (Wang et al., 2021). The latter is able to bind methylated histones by adopting a unique tandem Tudor-like fold (Law et al., 2013). SHH2 is encoded by a single gene in five grass species while by two genes in maize that have been functionally characterized (Wang et al., 2021) (**Figure 1C**). SHH1, the SHH2 paralog, interacts with Pol IV and is required for RNA-directed DNA methylation in *Arabidopsis* (Law et al., 2011, 2013), suggesting the different roles of SHH1 and SHH2 in regulating chromatin structure.

### 3.2 Characteristics of the SWI/SNF complex genes in sorghum

**Figure 2:**
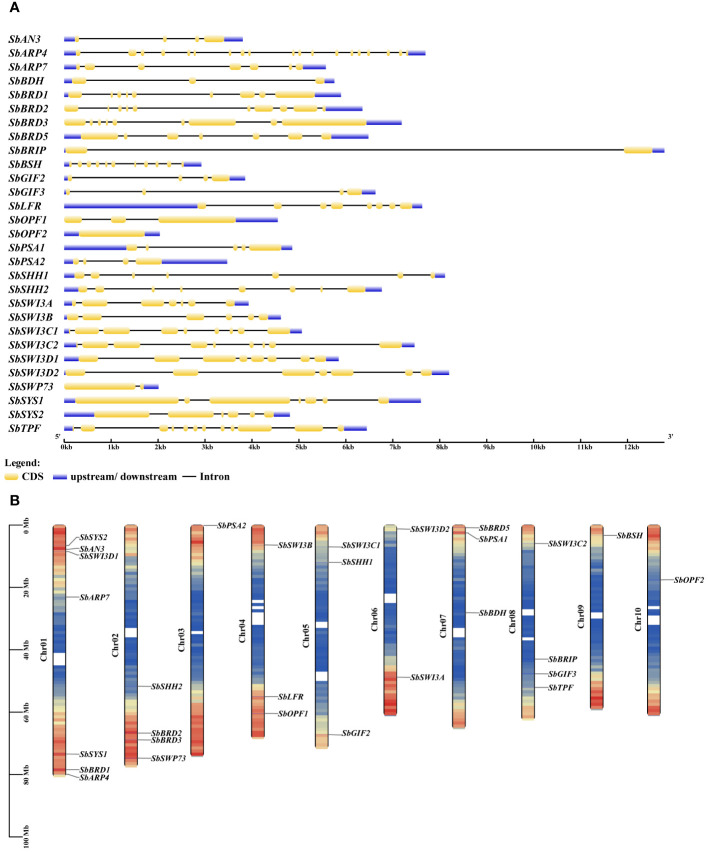
Gene structure **(A)** and chromosomal distribution **(B)** of the SWI/SNF complex genes in sorghum.

The sorghum genome contains 29 genes encoding the SWI/SNF complex subunits in addition to three Snf2 genes reported previously (**Table 1**) (Hu et al., 2022). The SWI/SNF complex subunits in sorghum are encoded by one gene with the exception of *SbSWI3C*, *SbSWI3D*, *SbGIF*, and *SbSYS*. The SWI/SNF complex genes excluding *SbARP4*, *SbARP7*, and *SbOPF2* possess at least one intron (**Figure 2A**). The number of introns ranges from one to 11. Notably, *SbBRIP* contains only one intron but as long as nearly 11 kb. All the SWI/SNF complex genes are distributed in 10 chromosomes of the sorghum genome (**Figure 2B**). *SbBDH*, *SbOPF2*, *SbSHH2*, *SbBRIP*, and *SbARP7* are located near the center of the chromosome where gene density is lower, while the others are located at the terminal of the chromosome with higher gene density. The isoelectric points (pI) of these subunits range from 4.68 to 9.66 (**Table 1**). The grand average of hydropathicity (GRAVY) ranges from −1.139 to −0.046 (**Table 1**). Subcellular localization prediction showed that most of the subunits were localized in the nucleus, implying the potential involvement of them in the formation of the SWI/SNF complex (**Table 1**).

### 3.3 Expression pattern of the SWI/SNF complex genes in sorghum

**Figure 3:**
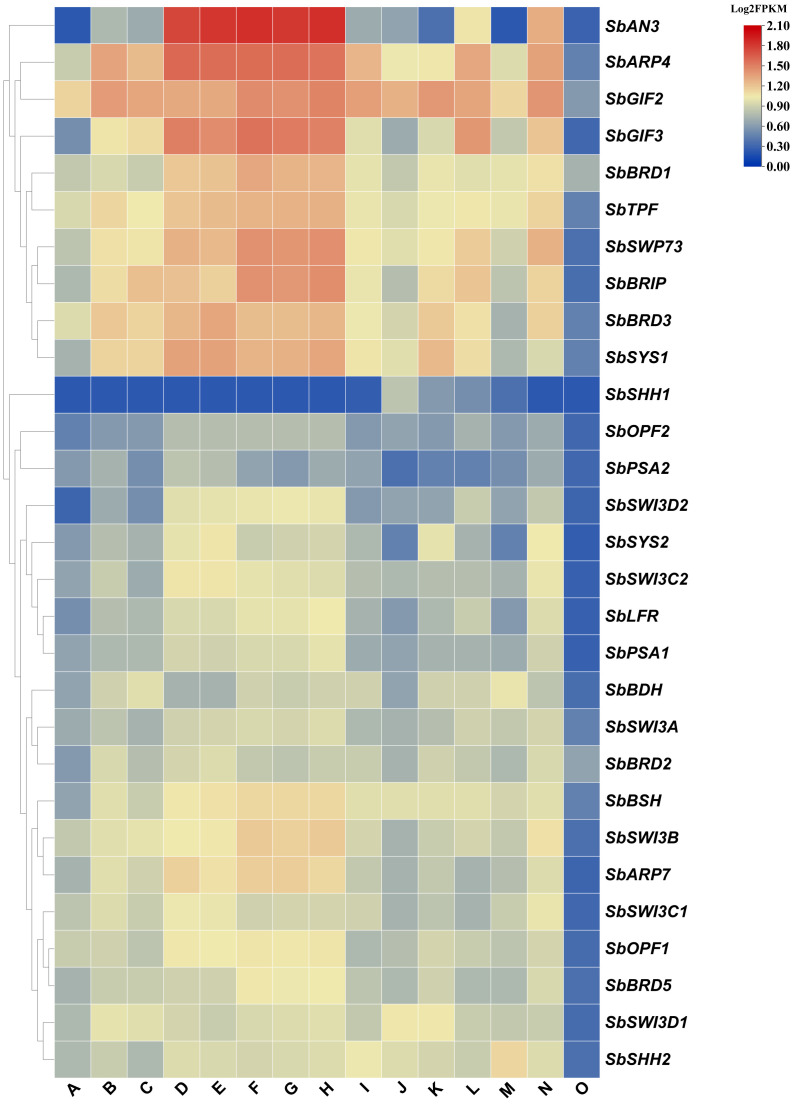
Expression pattern of the SWI/SNF complex genes in sorghum. **(A)** Leaf, **(B)** stem, **(C)** root, **(D)** vegetative meristem, **(E)** floral meristem, **(F)** inflorescence (1–5 mm), **(G)** inflorescence (1–10 mm), **(H)** inflorescence (1–2 cm), **(I)** spikelet, **(J)** endosperm (20 days after pollination), **(K)** pericarp (20 days after pollination), **(L)** embryo (20 days after pollination), **(M)** anther, **(N)** pistil, and **(O)** pollen (booting stage). The FPKM values for gene expression in each tissue were obtained from the Plant Expression ATLAS database. Subsequently, the Log_2_FPKM values of the SWI/SNF complex genes were computed and presented in a heatmap.

We collected expression data in 15 tissues of sorghum from the plant expression ATLAS database and investigated the expression profiles of the SWI/SNF complex genes. All the genes were expressed at a low level in pollen, suggesting that the chromatin remodeling activity is low and may not be required in the tissue (**Figure 3**). The expression profiles of these genes could be clustered into two groups (**Figure 3**). In one group, gene expression is higher on average, especially the expression levels of *SbAN3*, *SbGIF2*, *SbGIF3*, and *SbARP4* in the vegetative phase, floral meristems, and inflorescences. However, *SbAN3* was expressed at a very low level in leaves, pericarps, and anthers, suggesting a tissue-specific expression pattern of *SbAN3*, which is consistent with that of *Arabidopsis AN3*. *AN3* was mainly expressed in the basal region of leaf primordia but not in mature leaves and promoted leaf growth by regulating the cell proliferation process in *Arabidopsis* (Horiguchi et al., 2005). Similarly, the silencing of *BrAN3* in Chinese cabbage also led to early formation of the leafy head, and mutation of *MKB3* (*AN3* homolog in rice) reduced leaf size in rice (Shimano et al., 2018; Yu et al., 2019), suggesting the conserved function of *AN3* homologs in controlling plant leaf development. In the other group, we observed a lower expression level of genes in all the tissues, but most of them were constitutively expressed. The only exception is that *SbSHH1* was not expressed in most of the tissues but expressed in seeds including embryos, endosperm, and pericarps. By contrast, we detected the moderate expression of *SbSHH2* in all the tissues, making SbSHH2 instead of SbSHH1 a candidate subunit of the SWI/SNF complex.

### 3.4 *cis*-regulatory elements in the promoters of the SWI/SNF complex genes

**Figure 4:**
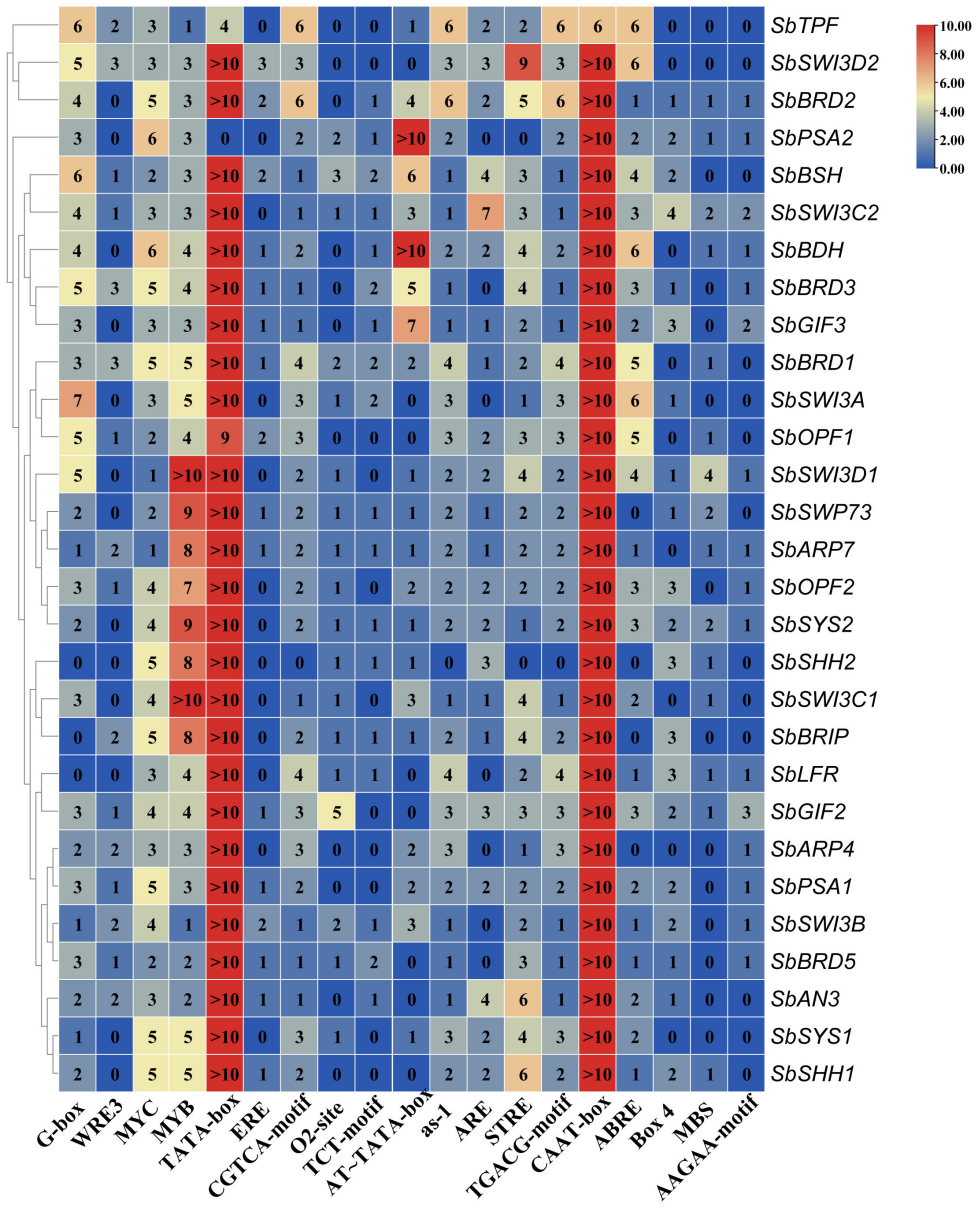
Analysis of *cis*-regulatory elements in the promoter of the SWI/SNF complex genes in sorghum.

To explore the potential regulatory mechanism of the SWI/SNF complex genes, we analyzed the promoter sequences (2,000 bp upstream of transcription start site) of these genes on the PlantCARE website. Interestingly, we found that multiple copies (>10) of TATA boxes and CAAT boxes were present in the promoter of nearly all the genes (**Figure 4**). The TATA box was considered the most universal *cis*-element in the core promoter and recognized by the TATA-binding box (TBP), which directs the assembly of the PIC. However, genome-wide surveys in various species revealed that the TATA box was only present in less than 50% of promoters (Savinkova et al., 2023), suggesting that it is required for the expression of a part of genes. It has been reported that TATA-containing genes are often highly responsive, while TATA-free genes are housekeeping genes in humans and yeast (Savinkova et al., 2023). CAAT box is bound by NF-Y family transcription factors that are important regulators of plant development and stress response (Myers and Holt, 2018). The common motifs shared by the majority of the SWI/SNF complex genes include G-box (bZIP G-box binding factors), MYC, MYB, CGTCA-motif, as-1, STRE, TGACG-motif, and ABRE (**Figure 4**). In addition, the motifs such as WRE3, ERE, O2-site, TCT-motif, AT-TATA box, ARE, Box4, MBS, and AAGAA-motif also exist in some of the SWI/SNF complex genes (**Figure 4**).

### 3.5 Transactivation activity of some of SWI/SNF complex subunits

**Figure 5:**
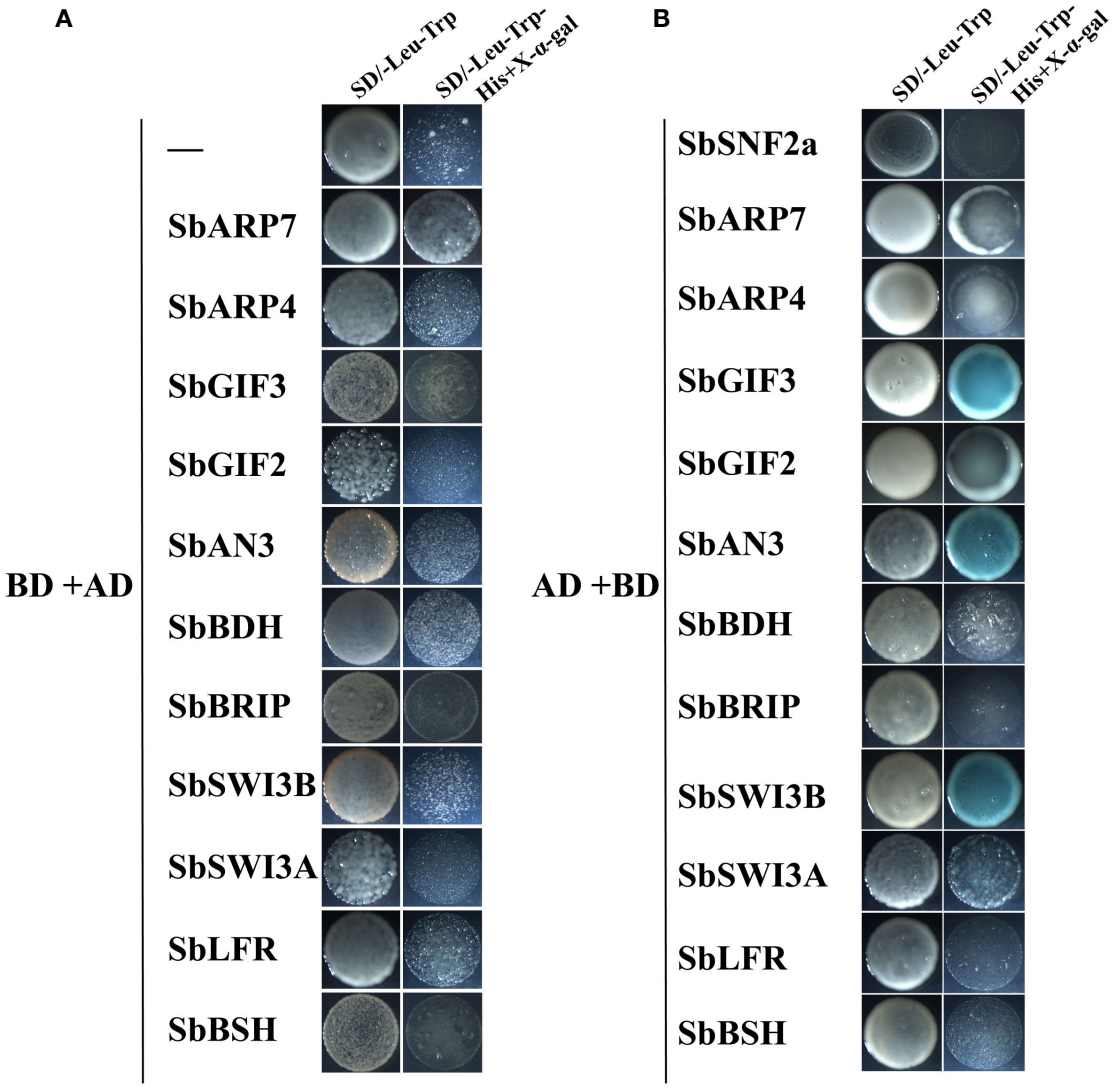
Detection of transactivation activity of sorghum SWI/SNF complex subunits in yeast. Twelve SWI/SNF complex genes in sorghum were inserted in pGADT7 vector and co-transformed into yeast (AH109) with pGBKT7 empty vector **(A)**, or inserted in pGBKT7 vector and co-transformed into yeast with pGADT7 empty vector **(B)**. The transformed yeast strains were grown on SD/-Trp-Leu medium and SD/-Trp-Leu-His+X-α-Gal medium.

To verify the interaction relationship between SWI/SNF complex subunits, we selected 12 genes for Y2H analysis. We inserted full-length cDNAs of these genes into pGADT7 and pGBKT7, and we co-transformed the recombinant vectors into yeast with the other empty vectors for the self-activation test. We found that yeast transformed with SbAN3, SbGIF3, SbSWI3B-pGBKT7, and pGADT7 vectors can grow and turn blue in SD/-Leu-Trp-His+X-α-Gal medium (**Figure 5B**), while the other transformants cannot grow (**Figures 5A, B**). This suggests that SbAN3, SbGIF3, and SbSWI3B can activate transcription in yeast. The transactivation activity of AN3 in *Arabidopsis* has also been reported (Kim and Kende, 2004). Additionally, the whole protein of AN3 is required for the transactivation activity, as the truncated proteins lose the ability to activate transcription. However, the lower activity of SbGIF2 in comparison with SbAN3 and SbGIF3 indicates that variation of some amino acids in SbGIF2 may affect the transactivation activity. Different from AtSWI3B, which is unable to activate transcription in yeast (Sarnowski et al., 2002), SbSWI3B obtained the transactivation activity also possibly due to variation of some key amino acids. By contrast, *Arabidopsis* LFR but not sorghum SbLFR can activate transcription in yeast (Lin et al., 2021). This indicates that the molecular function of some subunits has changed during evolution in grass species such as sorghum. The transactivation activity exhibited by these subunits in the SWI/SNF complex also suggests that the complex may have a role in recruiting the PIC in addition to facilitating PIC binding to the core promoter through chromatin remodeling.

### 3.6 Protein–protein interaction map of the SWI/SNF complex

**Figure 6:**
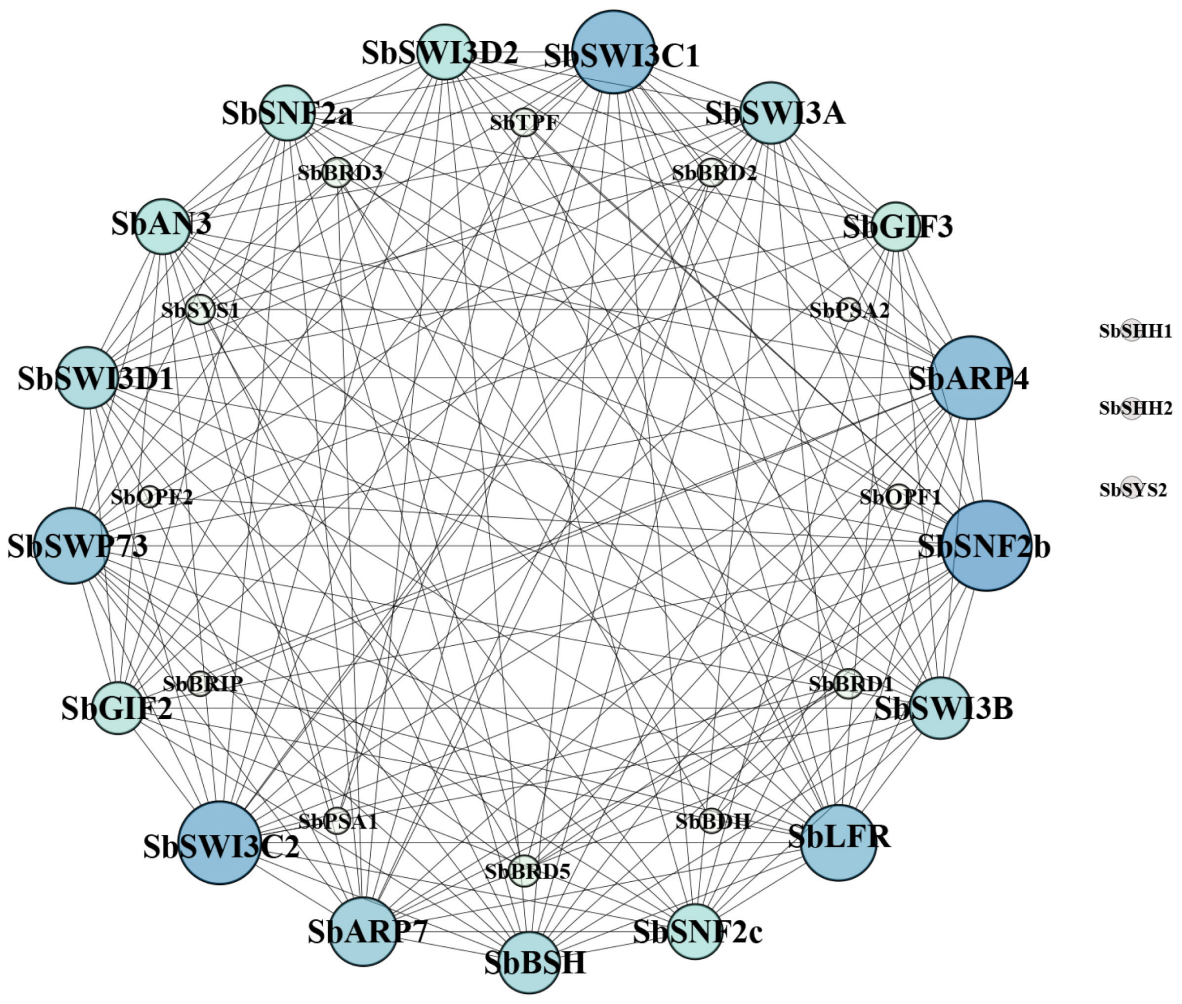
Interaction map of sorghum SWI/SNF complex subunits predicted in STRING database.

**Figure 7:**
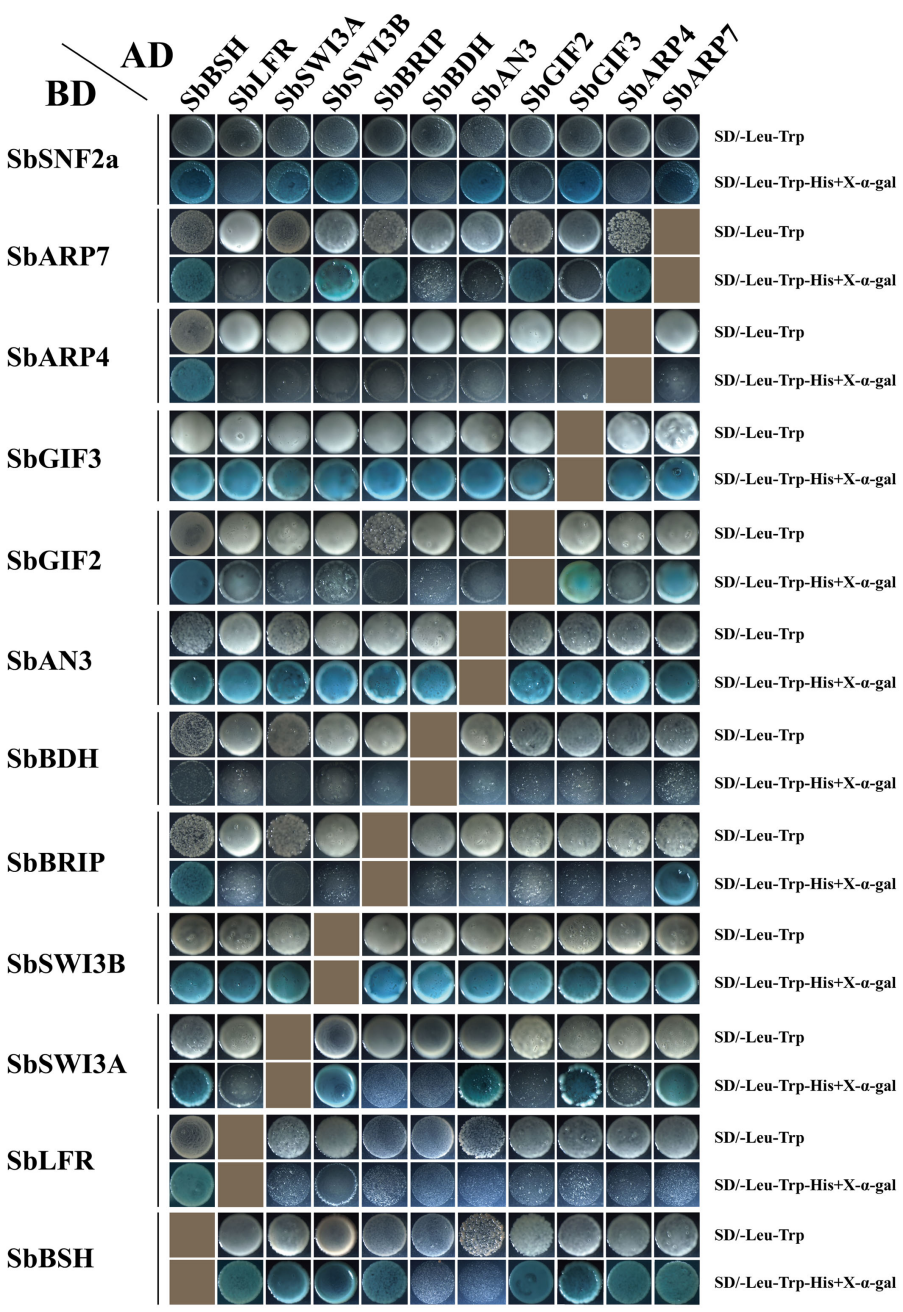
Interaction relationship of a part of SWI/SNF complex subunits confirmed by yeast two-hybrid assay. Twelve SWI/SNF complex genes in sorghum were inserted in pGADT7 and pGBKT7 vectors and co-transformed into yeast (AH109). The transformed yeast strains were grown on SD/-Trp-Leu medium and SD/-Trp-Leu-His+X-α-Gal medium.

The protein–protein interaction network of the SWI/SNF complex subunits in sorghum was established in the STRING database. The result showed that SbSHH1/2 and SbSYS2 were not involved in the interaction with the other subunits and thus were not in the interaction map (**Figure 6**). SbSWI3C1/2, SbARP4/7, SbSNF2c, SbLFR, and SbSWP73 interacted with more than half of the complex subunits, while SbBRD1/2/3/5, SbPSA1/2, SbSYS1, SbOPF1/2, SbBRIP, SbTPF, and SbBDH interacted with only a few subunits (**Figure 6**). The results confirmed by Y2H assays indicated that SbBSH interacted with most subunits including SbSNF2a (1–1360aa), SbARP4, SbARP7, SbGIF2, SbGIF3, SbBRIP, SbSWI3A, SbSWI3B, and SbLFR, which is not analogous to its ortholog in *Arabidopsis* AtBSH that interacted with three subunits and is only present in the MAS complex (**Figure 7**) (**Table 2**) (Guo et al., 2022). The interaction of SbBDH with the other selected subunits could not be detected by Y2H (**Figure 7**) (**Table 2**), which is similar to BCL7A/B in *Arabidopsis* (Guo et al., 2022). SbLFR interacted only with SbBSH, while OsLFR in rice and LFR in *Arabidopsis* interacted with at least three or four different subunits (**Figure 7**) (**Table 2**) (Qi et al., 2020; Guo et al., 2022). In *Arabidopsis*, BRM interacted with most of the BAS subunits by its N-terminal domain (Guo et al., 2022), but SbSNF2a (1–1360aa) (BRM homolog in sorghum) interacted with only six of 11 selected subunits (**Figure 7**) (**Table 2**). In addition, the interaction subunits of SbSWI3A/B, SbBRIP, SbAN3, SbGIF2, SbGIF3, SbARP4, and SbARP7 were different from their homologs in *Arabidopsis* (**Figure 7**) (**Table 2**). This suggests that the evolution of the subunits may lead to unique binding features of the proteins, resulting in the formation of distinct SWI/SNF complexes in sorghum. However, we found that SbSWI3A interacts with SbSWI3B to form a heterodimer as revealed in *Arabidopsis*, yeast, and humans (**Figure 7**) (**Table 2**) (Sarnowski et al., 2005), demonstrating that the dimerization of SWI3 proteins is also conserved in sorghum.

**Table 2:** Interaction relationship of SWI/SNF complex subunits in sorghum tested by Y2H.

	SbBSH	SbLFR	SbSWI3A	SbSWI3B	SbBRIP	SbBDH	SbAN3	SbGIF2	SbGIF3	SbARP4	SbARP7
SbSNF2a	+^a^	−^b^	+	+	−	−	+	−	+	−	+
SbARP7	+	−	+	+	+	−	−	+	−	+	
SbARP4	+	−	−	−	−	−	−	−	−		
SbGIF3	+	−	+	о^c^	−	−	о	+			
SbGIF2	+	−	−	−	−	−	−				
SbAN3	−	−	+	о	−	−					
SbBDH	−	−	−	−	−						
SbBRIP	+	−	−	−							
SbSWI3B	+	−	+								
SbSWI3A	+	−									
SbLFR	+										

Y2H, yeast two-hybrid.

a “+” Interaction.

b “−” Not interaction.

c “о” Not confirmed.

### 3.7 Dynamic change of nucleosome organization during sorghum development and stress response

**Figure 8:**
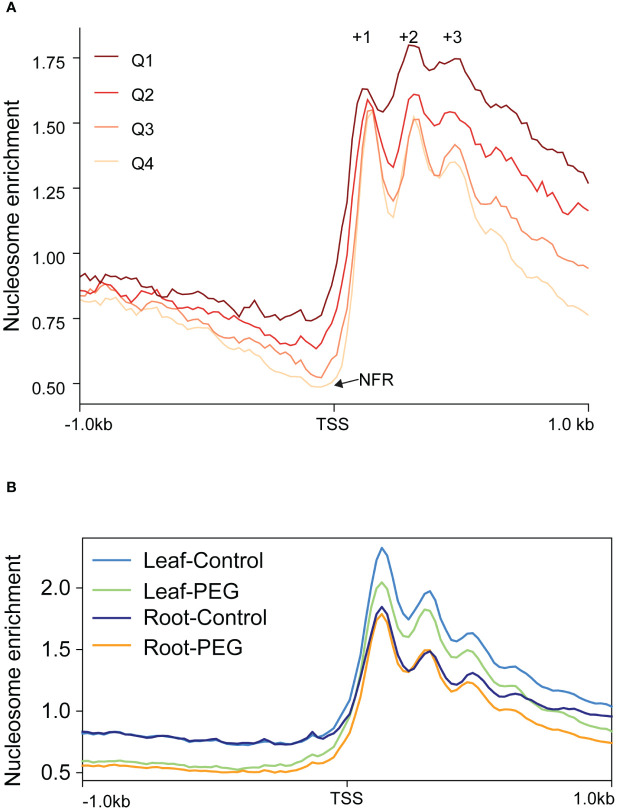
Features of nucleosome organization during sorghum development and stress response. **(A)** Metaplots of nucleosome profiles near TSS of all genes with different expression levels. All expressed genes (FPKM < 0.1, *n* = 24,718) were divided into four quartiles (Q1–Q4) by their expression level. Q1: 0.1 ≤ FPKM < 0.86 (*n* = 6,180), Q2: 0.86 ≤ FPKM < 5.95 (*n* = 6,179), Q3: 5.95 ≤ FPKM < 22.30 (*n* = 6,179), and Q4: 22.30 ≤ FPKM < 20610.17 (*n* = 6,179). The positions of +1 to +3 nucleosomes and the nucleosome-free region (NFR) are indicated. **(B)** Metaplots of nucleosome profiles near TSS of all genes in response to PEG treatment in leaves and roots.

As chromatin remodeling catalyzed by the SWI/SNF complex plays important roles in plant development and stress response (Song et al., 2021; Shang and He, 2022), we would like to understand the dynamic change of nucleosome organization during sorghum development and stress response. Therefore, we performed MNase-seq and RNA-seq to reveal the genome-wide nucleosome profile in sorghum. We found that different from five or more phased nucleosomes (a succession of nucleosomes is evenly spaced and well-positioned, as defined by Baldi, 2019) in rice and *Arabidopsis* (Li et al., 2014; Lu et al., 2020), there were only three peaks representing clearly phased nucleosomes (numbered as +1, +2, and +3) downstream from the TSS in sorghum (**Figure 8A**). Moreover, the peak indicating −1 nucleosome was not clear upstream from the TSS, although nucleosome-free region (NFR) did apparently exist (**Figure 8A**). In other words, the length of NFR is highly variable among different genes in sorghum. However, gene expression levels are positively associated with the depth of NFR and the level of nucleosome phasing downstream of the TSS, which is similar to rice and *Arabidopsis*.

Subsequently, genome-wide nucleosome profiles in response to PEG treatment in leaves and roots were analyzed. The result showed that the nucleosome level from TSS to downstream 1 kb in leaves was generally elevated compared to that in roots, while nucleosome phasing did not clearly change (**Figure 8B**). This indicates that nucleosome occupancy (indicating for each base pair in the genome the fraction of DNA molecules in the population that is actually occupied by a nucleosome, defined by Baldi, 2019) but not phasing is dynamic in the process of development, which may be involved in transcriptional regulation of specific genes. It has been reported that BRM regulates *Arabidopsis* vegetative phase change by decreasing nucleosome occupancy of +1 nucleosome at the *MIR156A* locus to activate its expression (Xu et al., 2016), reflecting histone eviction function of the SWI/SNF complex in a plant. A general decrease of nucleosome occupancy in response to PEG treatment was also observed in leaves, while a clear decrease of the nucleosome level was only observed before TSS and after phased nucleosomes in roots (**Figure 8B**), suggesting that the changes in chromatin structure in response to stress vary among different tissues. Similarly, in rice, nucleosome phasing remains unchanged in response to Pi starvation, while nucleosome occupancy changes, albeit differentially in coding and non-coding regions (Zhang et al., 2018). Moreover, in *Arabidopsis*, heat stress induces lower nucleosome occupancy, which is probably mediated by BRM, to activate gene expression. This demonstrates that the reduction in nucleosome occupancy to activate gene expression in response to stress is a common mechanism in plants.

## 4 Conclusions

In general, we observed conserved subunits of the SWI/SNF complex in grass species, similar to those in *Arabidopsis*. However, several subunits, including BSH, SWP73, LFR, BDH, TPF, and BRIP, are encoded by single-copy genes in grass species, whereas most of them are encoded by two copies in *Arabidopsis*. Some grass species, such as maize, sorghum, and rice, have undergone ancient whole-genome duplication (WGD) events (Paterson et al., 2004). It has been proposed that the return to a single gene copy after genome duplication could be explained by the gene balance hypothesis, where selection is based on deleterious unbalanced gene duplications among complex subunits (Garcia and Messing, 2017). Expression profile analysis in sorghum has revealed that these genes are highly expressed, suggesting that their single copy may support the function of the complex. Moreover, the interaction relationship of the subunits is different in sorghum from that in *Arabidopsis*. This suggests that distinct SWI/SNF complexes may be formed in sorghum, which requires further investigation by Affinity purification-mass spectrometry (AP-MS) experiments. In addition, we found that SbAN3, SbGIF3, and SbSWI3B had transactivation activity in yeast, which is not completely consistent with their homologs in *Arabidopsis*. Finally, epigenomic and transcriptomic analyses revealed the positive association of gene expression levels with nucleosome phasing, which is conserved in plants. However, the dynamic change in nucleosome occupancy prevailed during development and stress response. These results demonstrate that SWI/SNF complexes in sorghum may have evolved different characteristics and functions.

## Data availability statement

The datasets presented in this study can be found in online repositories. The names of the repository/repositories and accession number(s) can be found below: https://www.ncbi.nlm.nih.gov/, PRJNA952350.

## Author contributions

XC: Investigation, Writing – review & editing. CH: Data curation, Writing – review & editing. HX: Investigation, Writing – review & editing. GZ: Investigation, Writing – review & editing. QH: Writing – review & editing. ZD: Writing – review & editing. XQ: Writing – review & editing. XS: Funding acquisition, Supervision, Writing – original draft, Writing – review & editing. YH: Conceptualization, Funding acquisition, Project administration, Writing – original draft, Writing – review & editing.

## Supplementary material

Supplementary Figure 1 Domain architecture of the SWI/SNF complex subunits in six grass species.

Supplementary Figure 2 Sequence alignment of BSH proteins in six grass species.

Supplementary Figure 3 Sequence alignment of LFR proteins in six grass species.

Supplementary Figure 4 Sequence alignment of BDH proteins in six grass species.

Supplementary Figure 5 Phylogenetic tree of actin proteins in six grass species.

Supplementary Figure 6 Sequence alignment of PSA1 proteins in six grass species.

Supplementary Figure 7 Sequence alignment of PSA2 proteins in six grass species.

Supplementary Figure 8 Sequence alignment of SYS proteins in six grass species.

Supplementary Table 1 Primers used in this study.

Supplementary Table 2 List of SWI/SNF complex subunits in five grass species.
