# Supplementary material for: Characterization of the SWI/SNF complex and nucleosome organization in sorghum
Source: Front Plant Sci. 2024 Jun 26;15:1430467. doi: 10.3389/fpls.2024.1430467 (PMC11234113; doi:10.3389/fpls.2024.1430467)
Supplement: Supplementary Figure 2 — Sequence alignment of BSH proteins in six grass species. [file Image_2.pdf]

```
AtBSH      MKGLVSTGWKGPVKFRIYRPTAENLVPIRLDIQFEGQRYKDAFTWNPS.............................DPDNEVVIFAKRTVKDLKLPYAFVTQIAQSIQSQLSDFRAYEGQDMYTGEKIIPIKLDLRVNHTLIKDQFLW.   120
SbBSH      MKTVSLGAS.RPSTVNFRMPTRDNLVPIRVDVEVDGQRYRDAFTWNPR.............................DPDSEIISFAKRTAKDLKLPANFVPQMLQSIQGQLAEFRSYEGQEMQIKEKIVPLKIDLRVNNTVIRDQFLW.   119
OsBSH      ..............MRYRLLL...LLLLWVGCLLP..........................................HPDSEIISFAKRTAKDLKLPANFVPQMLHSIQGQLTEFRSYEGEEMQIREKIVPLKIDLRINNTVIRDQFLW.    90
ZmBSH      MKTVSLGAS.RPSTVNFRMPTRDNLVPIRVDVEVDGQRYRDAFTWNPR.............................DPDSEIISFAKRTAKDLKLPANFVPQMLQSIQGQLAEFRSYEGQEMQIKEKIVPLKIDLRVNNTVIRDQFLWV   120
BdBSH      MKTVSLSAPTRSSTVKFRMPTRDNLVPIRVDVEVDGQRYRDAFTWNPR.............................DPDSEIISFAKRTAKDLKLPASFVPQMLQSIQGQLAEFRSYEGQEMQIKEKIMPLKIDLRVNNTLVRDQFLW.   120
HvBSH      MKTVSLGAP.RSSTVKFRMPTRDNLVPIRVDIEVDGQRYRDAFTWNPRGTVPPPLPSLVLFGQVGRFLMVRSFLRRADPDSEVITFAKRTAKELKLPATFVPQMLHSIQGQLAEFRSYEGQEMQVKEKIMPLKIDLRVNNTIVRDQFLW.   148
SiBSH      MKTVSLGAS.RPSTVNFRMPTRDNLVPIRVDIEVDGQRYRDAFTWNPR.............................DPDSEIISFAKRTAKDLKLPANFVPQMLQSIQGQLAEFRSYEGQEMQIKEKIVPLKIDLRVNNTVIRDQFLW.   119
Consensus                          l                                                     pd e   fakrt k lklp  fv q   siq ql  fr yeg  m   eki p k dlr n t   dqflw

AtBSH      ................................DLNNFESDPEEFARTLCKDLGVEDPEVGPAVAFAIREQLYEIAIQSVASARESR.LSKKGRRGSDHGSASKASGLSMDLMKLFSFKSSVVRKRKDLDVYEPVVDLLTSEEVDALEARE   237
SbBSH      ................................DIGNLDSDPEEFARTLCDDLNITDPEVGPAIAVSIREQLYEIASQSVSVMREKQ.MSKKGRRAPEFSSNSKAVNNAVDLFKYFGSKGSVIRKRKEWYLYEPVVDVVASEEDGKEEVNN   236
OsBSH      ................................DIGNLDSDPEEFARTLCDDLNITDPEVGPAIAVSIREQLYEIASQSVSAMREAARVSKKGRRAPEFASNSKAMNNSLDLFKYFGSKGSVVRKRKEWYLYEPVVDVITNEEVGVTDATE   208
ZmBSH      CTCIVVIFGSPHVLIIALTPIYSTLLLPLGGQDIGNLDSDPEEFARTLCDDLNITDPEVGPAIAVSIREQLYEIASQSVSVMREKQ.MSKKGRRAPEFSSNSKAVNNAVDLFKYFGSKGSVIRKRKEWYLYEPVVDVVANDEDGKEEANN   269
BdBSH      ................................DIGNLDSDPEEFARTLCDDLNITDPEVGPAIAVSIREQLYEIASQSVSAMREAK.VSKK.RRAPEFASNSKAMNNAVDMFKYFGSKGSVIRKRKEWYLYAPVVDVVPNEEVVVVDTTE   236
HvBSH      ................................DIGNLESDPEEFARTLCDDLNITDPEVGPAIAVCIREQLYEIASQTVSAMREAK.MSKK.RRAPEFASNSKAMNNAVDMFKYFGSKGSVIRKRKEWYLYEPVVDVVPKEEAAVVDAKE   264
SiBSH      ................................DIGNLDSDPEEFARTLCDDLNITDPEVGPAIAVSIREQLYEIASQSVSVMREKQ.LSKKGRRAPEFSSNSKAVNNAVDLFKYFGSKGSVIRKRKEWYLYEPVVDVVANEEDGKEEPNN   236
Consensus                                  d  n  sdpeefartlc dl   dpevgpa a  ireqlyeia q v   re    skk rr     s ska     d  k f  k sv rkrk     y pvvd     e

AtBSH      ERHAR.............                                                                                                                                            242
SbBSH      SSRPKKRAEEEKVASLQS                                                                                                                                            254
OsBSH      EINSRCD...........                                                                                                                                            215
ZmBSH      SSRPKKRADEEKAASLQS                                                                                                                                            287
BdBSH      GA................                                                                                                                                            238
HvBSH      QLK...............                                                                                                                                            267
SiBSH      SSRPKKRAEEEKVASLQS                                                                                                                                            254
Consensus
```
