# Supplementary material for: Characterization of the SWI/SNF complex and nucleosome organization in sorghum
Source: Front Plant Sci. 2024 Jun 26;15:1430467. doi: 10.3389/fpls.2024.1430467 (PMC11234113; doi:10.3389/fpls.2024.1430467)
Supplement: Supplementary Figure 3 — Sequence alignment of LFR proteins in six grass species. [file Image_3.pdf]

```
AtLFR   ...MQKRELGKSGGNSGGSSGPPAKRGRPFGSTSA.NSAAAAAAAAAADAMSPSALLGPSLLVHNSFVEQNNRRIVLALQSGLKSEVTWALNTLTLLSFKEKEDIRRDVMPLAKIAGLLDALLLIIDDWRDIALPKDLTRGTRVRTLGTN    146
SbLFR   ....MQKQSGKSGGTGGGT...PAKRGRPFGSTTGGGAAAAAAAAAVVDPGASAALVGPSLQVLSALSDQNNKRIVLALQSGLKSEILWALNALTVLSFKEKDDQRRDTTPLAKVPGLLDALLQVIDEWRDISMPKDHLKPPRVRTLGAN    143
OsLFR   MSHVRSAPAGKSGGGGGST...PAKRGRPFGSTTGSGAAAAAAAAAIGDAAAPAALVGPSLQVLTALSDQNNKRIVLALQSGLKSEILWALNALTVLSFKEKDDLRRDTTPLAKVPGLLDALLQVIDDWRDIAMPKDHTKPPRVRTLGVN    147
BdLFR   ....MQKQTGKSGGSGGST...SAKRGRPFGSTTG.GAAAAAAAAAIGDPAAPAALVGPSLQVLTALSDQNNKRIVLALQSGLKSEILWALNALTVLSFKEKDDLRRDTTPLAKVPGLLDALLQVIDDWRDIAMPKDHTKPPRVRTLGVN    142
HvLFR   ....MQKQTGKSGGGGSS...AAKRGRPFGSTTGSGAAAAAAAAAVGDPAAPAALVGPSLHVLTALSDQNNKRIVLALQSGLKSEILWALNALTVLSFKEKDDLRRDTTPLAKVPGLLDALLQVIDDWRDIAMPRDHTKPPRVRTLGVN    143
SiLFR   ....MQKQTGKSGGSGGGT...PAKRGRPFGSTTGSGAAAAAAAAAVGDPGAPAALVGPSLQVLSALSDQNNKRIVLALQSGLKSEILWALNALTVLSFKEKDDFRRDTTPLAKVPGLLDALLQVIDEWRDIAMPKDHLKPPRVRTLGAN    143
ZmLFR   ....MQKQTGKSGGTGGGT...PAKRGRPFGSTTGGGAAAAAAAAAVVDPGAPAALVGPSLQVLSALSDQNNKRIVLALQSGLKSEILWALNALTVLSFKEKDDLRRDATPLAKVPGLLDALLQVIDEWSDISMPKDHTKPPRLRTLGAN    143
Consensus        gksgg  g      akrgrpfgst     aaaaaaaa  d     al gpsl v      qnn rivlalqsglkse  waln lt lsfkek d rrd  plak  glldall  id w di  p d     r rtlg n

AtLFR   ASVTGFGNEYDALASIQPPGSGIGSSAAEALGKKSTGKHQSSQWWMEEDGLFNLDDEGRSEKQMCAIAASNVIRNFSFMPDNEVVMAQHRHCLETVFQCIHDHMTEDEELVTNSLETIVNLAHLMDLRIFSSLKQSYININEKKAVQAVV    296
SbLFR   TTLSGFG..QENMEKVYSDTATTSNNDQSKTEDSSVTKKRAASFWFDEDGLFNNDDEGRAERQQCAIAASNIIRNFSFMPENEIIMVQHRHCLESIFHCLEDQNREDDELITNMVETLVNLAPVLDLRIFSSSKPSFIKMTEKGAVHAIM    291
OsLFR   TTLSGFG..HENVEKVYSDTTTPSD.DQTKTADSTVTKKRSAGFLFDEEGLFNVDDEGRTEKQQCAVAASNIIRNFSFMPENETVMVQHRHCLETVFQCLEDQNTEDDELITNMLETLVNLAPVLDLRIFSSSKPSFIKITEKRAVQAIM    294
BdLFR   TTLSGFG..LENIGKVYSDTATPSN.DESKKEDSTITKKRSAGFLLDEEGLFNMDDEGRTERQQCAVAASNIIRNFSFMPENETIMVQHRHCLETIFQCLEDQNTEDDELITNMLETLVNLAPVLDLRIFSSSKPSFIQMTEKSAVHAIM    289
HvLFR   TTISGFG..LENVGKVYSDSTTPPN.DQSKIEASTITKKRSAGFLFDEDGLFNIDDEGRTERQQCAVAASNIIRNFSFMPENETIMVQHRHCLETIFQCLEDQNAEDDELITNMLEALVNLAPVLDLRIFSSSKPSYIKMAEKSAVHAIM    290
SiLFR   TTLSGFG..QENMEKVYSDTGTTSN.DQSKTEESSVTKKRSASFWFDEDGLFNNDDEGRAERQQCAIAVSNIIRNFSFMPENETIMVQHRHCLETVFQCLEDQNREDDELVTNMLETLVNLAPVLDLRIFSSSKPSFIKMTEKGAVHAIM    290
ZmLFR   TTLSGFG..QENMEKVYSDTATTSN.DQSKAEDSSVTKKRAASFWFDEDGLFNNDDEGRAERQQCAIAASNIIRNFSFMPENEIIMVQHRHCLETIFHCLENQNREDDELITNMLETLVNLAPVLDLRIFSSSKPSFIKMTEKGAIHAIM    290
Consensus   gfg                              k         e glfn ddegr e q ca a sn irnfsfmp ne  m qhrhcle  f c      ed el tn  e  vnla   dlrifss k s i   ek a  a

AtLFR   GILNSSVKAWNCAAAELLGRLIINPDNEPFISPLIPQIHKRLIDLLSIQAVDAQAAAVGALYNLVEVNMDCRLKLASERWAVDRLLKVIKTPHPVPEVCRKAAMILENLVSEPQNRGLLLAYENAFAELLFQEGKYSDSFARILYELTAR    446
SbLFR   GMLSSSVKPWHCAAAELIGRLIINPDNEPFLLPVIPQIYKRLVDLLSVPAYDAQAAAVSALYNVAEVNMDCRLKLASERWAVDRLLKIVKTPHPVPEVCRKTSMILESLVSEPQNRMHLLVHENTFAEILTSEGKYSDTFARILYELTAR    441
OsLFR   GMLASSIRVWHCAAAELIGRLIINPDNEPFLLPAIPQIYKRLVDLLSVPAVDAQAAAISALYNVAEVNMDFRLKLASERWAVDRLLKVVKTPHPVPEVCRKASMIVESLVSEPQNRMHLLVHENTFAEILTSEGKYSDTFARILYELTAR    444
BdLFR   GMLASSVKAWHCAAAELIGRLIINPDNESFLLPVISQIYKRLVDLLSVPAFDAQAAAVSALYNVSEVNMDCRLKLACERWAIDRLLKIVKAPHPVPEVCRKATVILESLVSEPQNKMHLLVHENTFAEILTTEGKYSDTFARILYELTAR    439
HvLFR   GMLASSIKAWHCAAAELIGRLIINPDNESFLVPVISQIYRRLVDLLSVPAFDAQAAAVSALYNVSEVNMDCRLKLASERWAVDRLLKIIKAPHPVSEVCRKAAVILESLVSEPQNRMHLLVHENTFAEILTSEGKYSDTFARILYELTAR    440
SiLFR   GMLSSSVKPWHCAAAELIGRLIINPDNESFLLPVIPQIYKRLVDLLSMPAYDAQAAAVSALYNVAEVNMDCRLKLASERWAVDRLLKIVKTPHPVPEVCRKTSMILESLVSEPQNRMHLLVHENTFAEILTTEGKYSDTFARILYELTAR    440
ZmLFR   GMLSSSVKPWHCAAAELIGRLIINPDNEPFLLPIIPQIYKRLVDLLSVPAYDAQAAAVSALYNVAEVNMDCRLKLASERWAVDRLLKIVKTPHPVPEVCRKTSMILESLVSEPQNRMHLLVHENTFAEILTSEGKYSDTFARIVYELTAR    440
Consensug l ss   w caaael grliinpdne f  p i qi  rl dlls  a daqaaa  alyn  evnmd rlkla erwa drllk  k phpv evcrk   i e lvsepqn   ll  en fae l  egkysd fari yeltar

AtLFR   SNSRVASARGIWGM.    460
SbLFR   PSNKVTSGQAIWGNI    456
OsLFR   PSNKVTAGQAIWGNI    459
BdLFR   PSNKVTSGQAIWGNI    454
HvLFR   PSNKVTSGQAIWGNI    455
SiLFR   PSNKGTSGQAIWGNI    455
ZmLFR   PSNKVTSGHAIWGNI    455
Consensus         iwg
```
