# Supplementary material for: Characterization of the SWI/SNF complex and nucleosome organization in sorghum
Source: Front Plant Sci. 2024 Jun 26;15:1430467. doi: 10.3389/fpls.2024.1430467 (PMC11234113; doi:10.3389/fpls.2024.1430467)
Supplement: Supplementary Figure 4 — Sequence alignment of BDH proteins in six grass species. [file Image_4.pdf]

```
AtBDH1    MEGVGARLGRSSTRYG...PATVFTGPVRKWKKKWVHVSPSSKKDNNNSSSGSAAAAASVVNGGSNSDGSNG.SHLLLYKWAPLSQNGNGNEDGKSESNSPSEDTVATVAEDPP.....RRRFKYVPIAVLEEQKKEITEIEEDDKIEED   141
AtBDH2    MEGVGSRLSRTSSRYSGPAATAVFSGRVRKWKKKWVRVSTSS.........VGVFRASKSNGRNNSNNSNSPHHLLLHKWTPLT..........SATVTASDANGSGETEESP.....KRRFRYAPIAMLEHREK...VISKDSEIEET   122
OsBDH     MEGVGARLGRTSARYG...TTTTFTGPVRKWRKDWVPVAAAA..........AAAAAAASAASST.AGAGSRGNNLVLFKWTPMN........GANGNGGGDGDQAAAAAAAEEEKATKRRRYVPVSVVEDERQE.SGKSDDENKAND   127
ZmBDH     MEGVGARLGRTSARYG...PATTFTGHVRKWRKEWVPVVAAAAAAA..ANANTSANGGTTSSTGMGSGGGSRGNNLLLFKWTPVN........GANGGGG..DGEQQQQ.AETA.....TRRRRYVPVSLMEEQRQE.STKSDDENKAND   128
BdBDH     MEGVGARLGRSSARYG...PATTFTGPVRKWRKEWVPIAAAA..........ATAASAATSST....AAGSRGNNLVLFKWTPLN........GANGGAGEGDGEQAAAAAETA.....TRRRRYVPVSIVEDQRQE.SAKSDDENKAND   119
HvBDH     MEGVGARLGRSSARYG...PAMTFTGPVRKWRKEWVPISAAT..........ATASSAGT.........GSRGNNLILFKWTPFN........GSN....EGD.EEQTAPAKTA.....TRRRRYVPVSVVQDQRQE.SAKSDDENKAND   109
SiBDH     MEGVGARLGRTSARYG...PATTFTGPVRKWHKEWVPVAAAAAN....ANTSASANGSAASSTGTGSGSGSRGNNLLLFKWTPVN........GANGGGGGSDGEQQAAAADTA.....TRRRRYVPVSVVEEQRQE.SAKSDDENKAND   129
SbBDH     MEGVGARLGRTSARYG...PATTFTGPVRKWHKEWVPVVAAA......VNTNASANGGAAPSTGTGSGGGSRGNNLLLFKWTPVN........GANGGGG..EGEQQQA.AETA.....TRRRRYVPVSLMEEQRQE.STKSDDENKAND   124
Consensus megvg rl r s ry        f g vrkw k wv                                  l l kw p                                  r y p                   d

AtBDH1    DKIDEDNKVEQEDKVDEDKTVEESSEKKAEVEVEEKPDINDVPMEDIQQVEEKIVQDDEEKVVRQDLNESTVDLGLNLNANDADAENDPKEDKPLE   237
AtBDH2    EEFDTESPLPKAVELDMNLTDSDQTKEAKTGNLNLGLCLNSEGTEE................................................   168
OsBDH     GEPSSAETEPSNGKTNINDTPMDESQASDE.ARYSGKNGGGTDLNLNLGLKDPDGDNDIDTEEQD....AENNTHTEN.RLKRKSVAPDLEMRM..   215
ZmBDH     GDPSSNETEPSNGKTDINDTPMDESQATDEDGRDSGKNGGGTDLNLNLGLKDPDGDNEVETSEKH...VAANNPQTEN.RSKRKSVTPDLEMTM..   218
BdBDH     GDPSSTETEPSNGKTNIDDTPMDESQASDE.ARDSGNNGGGTDLNLNLGPKDPDDEDEGDTGE.......QNEARTEH.RLKRKSVTPDLEMRM..   204
HvBDH     GEPSSTETEQSIGKTNIDDILMDESQASDE.VRDSGNFVGGTDLNLNLGLKDPDVEDEGDTVEDEGDTVEHHRVRTEH.RLKRKSVTPDLEMRM..   201
SiBDH     GDPSPNETEASNGKTDINDTPMDESQASDEDARDSGKNGGGTDLNLNLGLKDPDGDNEADTAEQQ...EAAKNPQTENNRFKRKSVTPDLEMRM..   220
SbBDH     GDPSSNETEPSNEKTDINGTPMDESQASDEDGRNSGKNGGETDLNLNLGLKDPDGDNEVETTEKH...EAANNPLTEN.RSKRKSVTPDLEMRM..   214
Consensus
```
