# Supplementary figures and images for: Characterization of the SWI/SNF complex and nucleosome organization in sorghum
Source: Front Plant Sci. 2024 Jun 26;15:1430467. doi: 10.3389/fpls.2024.1430467 (PMC11234113; doi:10.3389/fpls.2024.1430467)

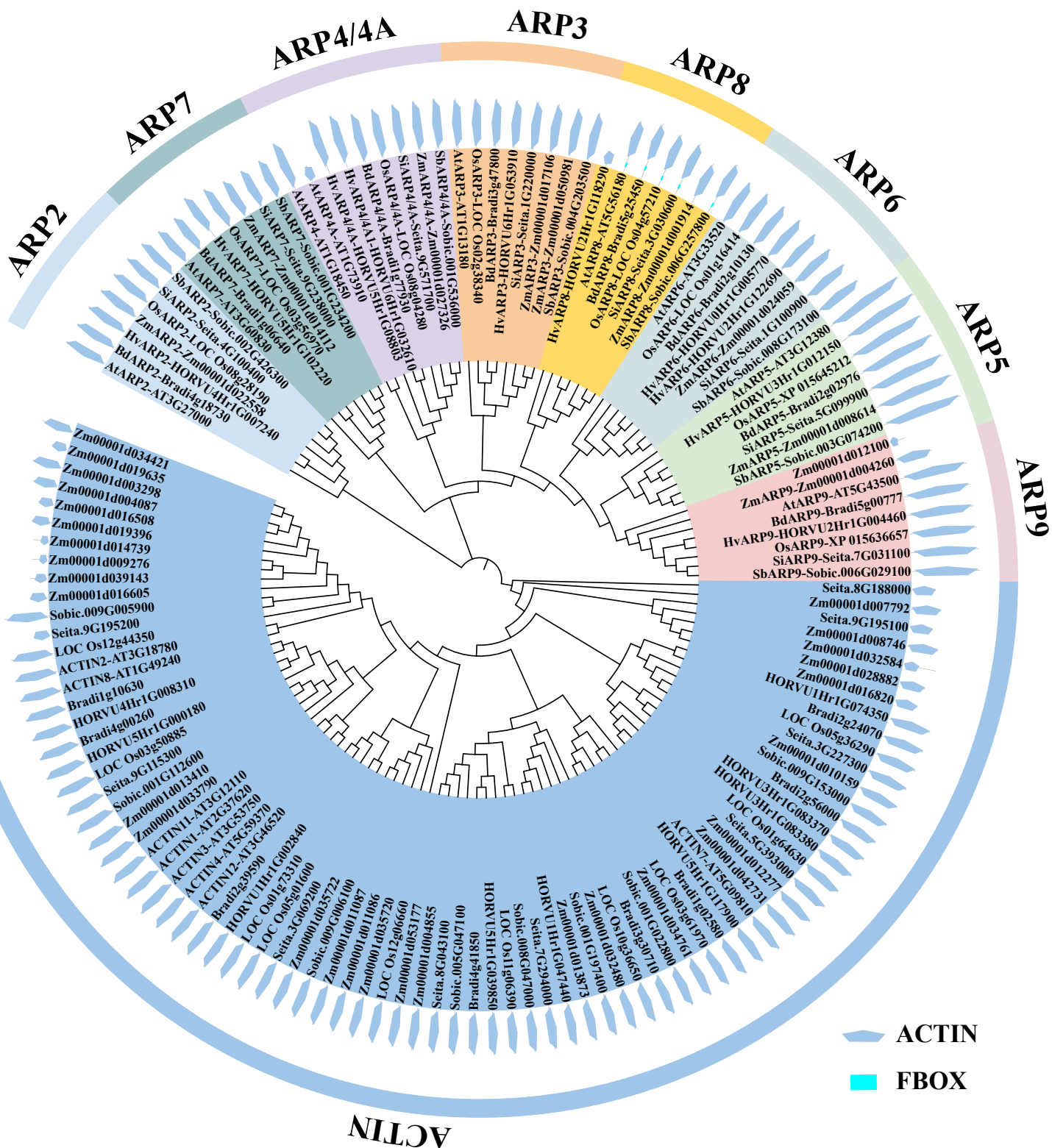

Supplement: Supplementary Figure 5 — Phylogenetic tree of actin proteins in six grass species. [file Image_5.pdf]
