# Supplementary material for: Characterization of the SWI/SNF complex and nucleosome organization in sorghum
Source: Front Plant Sci. 2024 Jun 26;15:1430467. doi: 10.3389/fpls.2024.1430467 (PMC11234113; doi:10.3389/fpls.2024.1430467)
Supplement: Supplementary Figure 6 — Sequence alignment of PSA1 proteins in six grass species. [file Image_6.pdf]

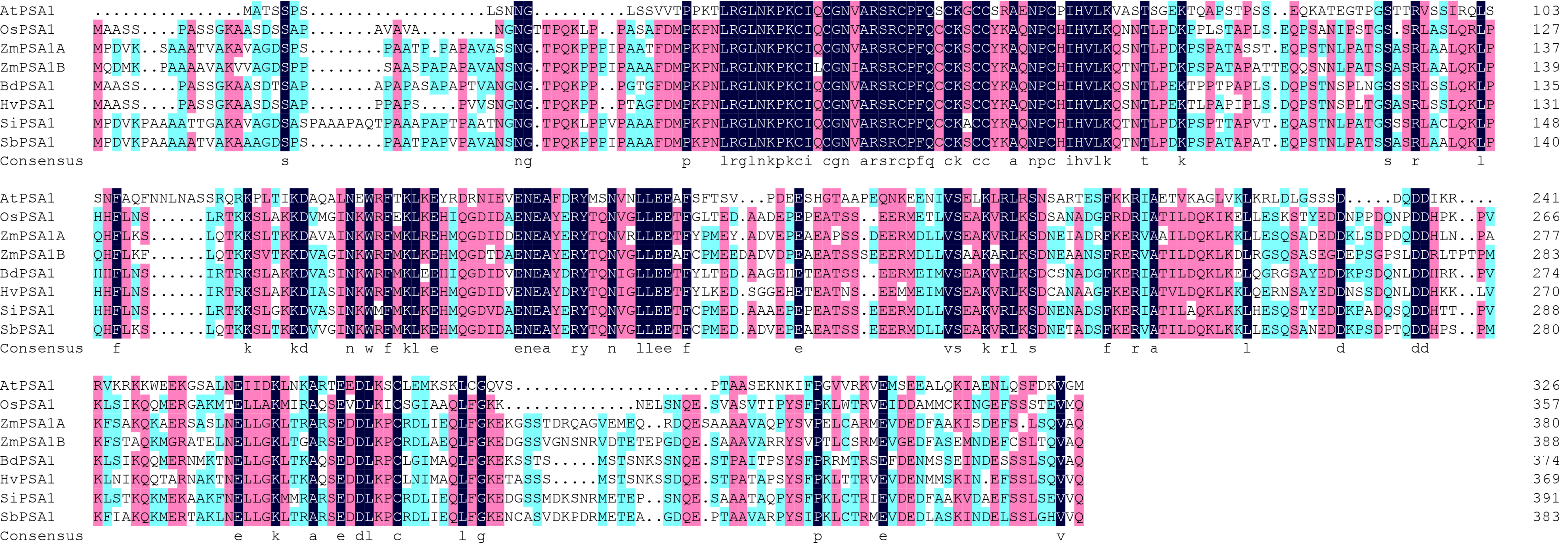
AtPSA1 ................MATSSPS..................LSNNG..........LSSVVTPPKTLRGLNKPKCIQCGNVARSRCPFQSCKGCCSRAENPCPIHVLKVASTSGEKTQAPSTPSS..EQKATEGTPGSTTRVSSIRQLS 103
OsPSA1 MAASS....PASSGKAASDSSAP.......AVAVA........NGNGTTPQKLP..PASAFDMPKPNLRGLNKPKCIQCGNVARSRCPFQCCKSCCYKAQNPCHIHVLKQNNTLPDKPPLSTAPLS.EQPSANIPSTGS.SRLASLQRLP 127
ZmPSA1A MPDVK..SAAATVAKAVAGDSPS........PAATP.PAPAVASSNG.TPQKPPPIPAATFDMPKPNLRGLNKPKCIQCGNVARSRCPFQCCKSCCYKAQNPCHIHVLKQSNTLPDKPSPATASST.EQPSTNLPATSSASRLAALQKLP 137
ZmPSA1B MQDMK..PAAAAVAKVVAGDSPP........SAASPAPAPAVANSNG.TPQKPPPIPAAAFDMPKPNLRGLNKPKCILCGNIARSRCPFQCCKSCCYKAQNPCHIHVLKQTNTLPDKPSPATAPATTEQQSNNLPATSSASRLAALQKLP 139
BdPSA1 MAASS....PASSGKAASDTSAP.......APAPASAPAPTVANGNG.TPQKPP..PGTGFDMPKPNLRGLNKPKCIQCGNVARSRCPFQCCKSCCYKAQNPCHIHVLKQSNTLPEKTPPTPAPLS.DQPSTNSPLNGSSSRLSSLQKLP 135
HvPSA1 MAASS....PASSGKAASDSSAP.......PPAPS....PVVSNGNG.TPQKPP..PTAGFDMPKPNLRGLNKPKCIQCGNVARSRCPFQCCKSCCYKAQNPCHIHVLKQSNTLPEKTLPAPIPLS.DQPSTNSPLTGSASRLSSLQKLP 131
SiPSA1 MPDVKPAAAATTGAKAVAGDSASPAAAPAQTPAAAPAPTPAATNGNG.TPQKLPPVPAAAFDMPKPNLRGLNKPKCIQCGNVARSRCPFQCCKACCYKAQNPCHIHVLKQTNTLPDKPSPTTAPVT.EQASTNLPATGSSSRLACLQKLP 148
SbPSA1 MPDVKPAAAAATVAKAAAGDSPS........PAATPAPVPAVANSNG.TPQKPPPIPAAAFDMPKPNLRGLNKPKCIQCGNVARSRCPFQCCKSCCYKAQNPCHIHVLKQTNTLPDKPSPATAPAT.EQPSTNLPATSSASRLAALQKLP 140
Consensus s ng p lrglnkpkci cgn arsrcpfq ck cc a npc ihvlk t k s r l
AtPSA1 SNFAQFNNLNASSRQRKPLTIKDAQALNEWRFTKLKEYRDRNIEVENEAFDRYMSNVNLLEEAFSFTSV...PDEESHGTAAPEQNKEENIVSELKLRLRSNSARTESFKKRIAETVKAGLVKLKKRLDLGSSSD.....DQDDIKR.... 241
OsPSA1 HHFLNS......LRTKKSLAKKDVMGINKWRFEKLKEHIQGDIDAENEAYERYTQNVGLLEETFGLTED.AADEPEPEATSS..EERMETLVSEAKVRLKSDSANADGFRDRIATILDQKIKELLESKSTYEDDNPPDQNPDDHPK..PV 266
ZmPSA1A QHFLKS......LQTKKSLTKKDAVAINKWRFMKLREHMQGDIDDENEAYERYTQNVRLLEETFYPMEY.ADVEPEAEAPSS.DEERMDLLVSEAKVRLKSDNEIADRFKERVAAILDQKLKKLLESQSADEDDKLSDPDQDDHLN..PA 277
ZmPSA1B QHFLKF......LQTKKSVTKKDVAGINKWRFMKLKEHMQGDTDAENEAYERYTQNVGLLEEAFCPMEEDADVDPEAEATSSSEEERMDLLVSAAKARLKSDNEAANSFRERVATILDQKLKDLRGSQSASEGDEPSGPSLDDRLTPTPM 283
BdPSA1 HHFLNS......IRTRKSLAKKDVASINKWRFMKLEEHIQGDIDVENEAYDRYTQNIGLLEETFYLTED.AAGEHETEATSS..EERMEIMVSEAKVRLKSDCSNADGFKERIATILDQKLKELQGRGSAYEDDKPSDQNLDDHRK..PV 274
HvPSA1 HHFLNS......IRTRKSLAKKDIASINKWRFMKLKEHMQGDIDVENEAYDRYTQNIGLLEETFYLKED.SGGEHETEATNS..EEMMEIMVSEAKVRLKSDCANAAGFKERIATVLDQKLKKLQERNSAYEDDNSSDQNLDDHKK..LV 270
SiPSA1 HHFLNS......LRTKKSLGKKDVASINKWMFMKLKEHMQGDVDAENEAYERYTQNVGLLEETFCPMED.AAAEPEPEATSS.EEERMDLLVSEAKVRLKSDNENADSFKERIATILAQKLKKLHESQSTYEDDKPADQSQDDHTT..PV 288
SbPSA1 QHFLKS......LQTKKSLTKKDVVGINKWRFMKLKEHMQGDIDAENEAYERYTQNVGLLEETFCPMED.ADVEPEAEATSS.EEERMDLLVSEAKVRLKSDNETADSFKERVATILDQKLKKLLESQSANEDDKPSDPTQDDHPS..PM 280
Consensus f k kd n w f kl e enea ry n llee f e vs k rl s f r a l d dd
AtPSA1 RVKRKKWEEKGSALNEIIDKLNKARTEEDLKSCLEMKSKLCGQVS....................PTAASEKNKIFPGVVRKVEMSEEALQKIAENLQSFDKVGM 326
OsPSA1 KLSIKQQMERGAKMTELLAKMIRAQSEVDLKICSGIAAQLFGKK.............NELSNQE.SVASVTIPYSFPKLWTRVEIDDAMMCKINGEFSSSTEVMQ 357
ZmPSA1A KFSAKQKAERSASLNELLGKLTRARSEDDLKPCRDLIEQLFGKEKGSSTDRQAGVEMEQ..RDQESAAAAVAQPYSVPELCARMEVDEDFAAKISDEFS.LSQVAQ 380
ZmPSA1B KFSTAQKMGRATELNELLGKLTGARSEDDLKPCRDLAEQLFGKEDGSSVGNSNRVDTETEPGDQE.SAAAVARRYSVPTLCSRMEVGEDFASEMNDEFCSLTQVAQ 388
BdPSA1 KLSIKQQMERNMKTNELLGKLTKAQSEDDLRPCLGIMAQLFGKEKSSTS.....MSTSNKSSNQE.STPAITPSYSFPRRMTRSEFDENMSSEINDESSSLSQVAQ 374
HvPSA1 KLNIKQQTARNAKTNELLGKLTKAQSEDDLKPCLNIMAQLFGKETASSS.....MSTSNKSSDQE.STPATAPSYSFPKLTTRVEVDENMMSKIN.EFSSLSQVVQ 369
SiPSA1 KLSTKQKMEKAAKFNELLGKMMRARSEDDLKPCRDLIEQLFGKEDGSSMDKSNRMETEP..SNQE.SAAATAQPYSFPKLCTRIEVDEDFAAKVDAEFSSLSEVVQ 391
SbPSA1 KFIAKQKMERTAKLNELLGKLTRARSEDDLKPCRDLIEQLFGKENCASVDKPDRMETEA..GDQE.PTAAVARPYSIPKLCTRMEVDEDLASKINDELSSLGHVVQ 383
Consensus e k a e dl c l g p e v
