# Supplementary material for: Characterization of the SWI/SNF complex and nucleosome organization in sorghum
Source: Front Plant Sci. 2024 Jun 26;15:1430467. doi: 10.3389/fpls.2024.1430467 (PMC11234113; doi:10.3389/fpls.2024.1430467)
Supplement: Supplementary Figure 7 — Sequence alignment of PSA2 proteins in six grass species. [file Image_7.pdf]

```
AtPSA2      ...................................................................................................................................................... 0
OsPSA2      ..........MCAAPMASTAQPQQQPQQQQEQQPVAAAAVP.TPAPPASEAQPQKPTRVSLSYEEISKLFSLPIAEAASILGVCTSVLKRICRSHGIVRWPYRKLVSGKSGDDTKNAEREKAKGLLEISKVAKQKALSASGLSTVSPGAF 139
ZmPSA2      ........................................MASDAPAEQPATQQKPTRVSLSYEEISKLFSLPIAEAASILGVCTSVLKRICRTHGIVRWPYRKLVSGKAGDDTKGPDSDKANELLEVSKIAKQKAPSASGPSVVSSSTS 110
BdPSA2      ..........MQKRPMAADAQAQQLPLEQAAAAVQAPVAP.........APAQPKPTRVSLSYEEISKLFSLPIAEAASILGVCTSVLKRICRTHGIVRWPYRKIVSGKTGDDVKNAEREKAKELLELSKIAKQKASP.........GGL 122
HvPSA2      ..........MSAAPMASNAQEQPPAQQQQAAAVQPSLALALVPQAQAQAQAQIKPTRVSLSYEEISKLFSLPIAEAASILGVCTSVLKRICRTHGIVRWPYRKIVSGK.GDDVKNAEREKAMQLLELSKIAKQKAISSSGLSTTSSGAF 139
SiPSA2B     ............................................MASNAQVQQPKPTKVSLSYEEISKLFSLPIAEAASILGVCTSVLKRICRSHGIVRWPYRKLVSGKAGDDTKGPEREKAKELLELSKIAKQKALSPSGSSIVSSSTS 106
SiPSA2A     MKNGNQTRNHHRAGRIRAAAVLLRRRRLLFDSPLQITAEQRSGGMASDAQAQQPKPTRVSLSYEEISKLFSLPIAEAASILGVGTSVLKRICRTHGIVRWPYRKLVSAKTGDDTKGPEREKAKELPELSKIAKQKASNSSASPIVSSTTS 150
SbPSA2      ........................................MASDAPTQQPAPQQKPTRVSLSYEEISKLFSLPIAEAASILGVCTSVLKRICRTHGIVRWPYRKLVSGKAGDDTKGPDRDKAKELLELSKIVKQKASSASGPSIVSSSTS 110
Consensus

AtPSA2      MMHSTVQHGGNKSGK............SNVWANTNLAKTVAA.VDEFKFGFPSGGLTTVSNKWWG........RAEKGGREDGG..................GEN....TENGHVAACDETQNSLVAIRKRIAEEGREAVELGLHQGFGS 107
OsPSA2      QGVAKSQQGSSKAGQVSPPGKQNVLGGSAILSYGTQTKGIPTYMDDFKYGFPSSGLSLQTMKWWGTDSHTETTPAKDDNGEAPESAN.EASKGMT.DDELDWGADEAEAEADADSAITTEPSAQLCSLRRKAVDDGRKLLTGKSCGGLEL 287
ZmPSA2      QGAAKSQQGNSKAGQFSVSPPTGKHNASLSLTH.SQAKAIPCYMDDFKYGFPSSGLSCETMKWWGTSSDTDYVPTKDGSHEPHESTTHEPSKGMTDDDELDWGAD..EAEAEADGTVTAEASAQLCSLRRKAVDDGRKLLNGHNRRGQEF 257
BdPSA2      QGVPKSQQGSTKAG..............PSLAHVSQAKDIPTYMDDFKNGFPSSGLSCETMKWWGASSHTETASAKGDSREAPESTN.EASKGMT.DDELDWGADEAEADTDGVVTS..EPSAQLCSLRRKAAGDGRRLLNGDTGRGQGF 254
HvPSA2      QAVSKAQQGSAKAGSAIGRQN......VPSLSQFSQAKDIPTYMDDFKYGFPSSGLSTETMKWWATDSHTETVAVKDDNREGSESTN.EASKGMT.DDELDWGADEPEADADGMDAAT.DPSAQLCSLRRKAAGDGRRLLNGDTGRVQQL 280
SiPSA2B     QGAAKSQQGSSKAGQVSVSPPAGKQNASPSLAHGSQTKAIPGYMDDFKYGFPSSGLPCETMKWWGGSSDTDCELTKDGNREAHG....EASKGMTDDDELDWGAD..EGEAEADGAVTAEASGQLCSLTRKAVDDGRKLLNGGTRRGREF 250
SiPSA2A     QGAAKSQQGSSKAGQVSVSPPAGNQNMSPSLAHGSQAKAIPSYMDDFKYGFPSSGLSCGTMKWWGGSSDADCAPTKDGSREAHG....EASKGMTEDDELDWGAD..EAETEADGAVTAEASAQLCSLRRKAVDDGRKLLNGDTRRGQEF 294
SbPSA2      QGAAKSQQGSSKAGQISVSPPAGKQNTSPSLAHGSQAKAIPSYMDDFKYGFPSSGLSCETMKWWGTSSDTDCVPAKDGSHEAHDLTTHEPSKGMTDDDELDWGADEAEAEAEADGAVTAEASAQLCSLRRKAVDDGRKLLNGGNRRGQEF 260
Consensus         q g  k g                       k      d fk gfps gl     kww                e                     g                     l          gr

AtPSA2      KRPGKRDQALLFQIFNSAMPKDWVTPDS 135
OsPSA2      CRLNKRQKMALAQVFGASLPEQLRSKLG 315
ZmPSA2      SRLNKRQKTALAQVFGASLPECCITRV. 284
BdPSA2      CRLNKRQKIALAQVFGASLPEQWSSKLA 282
HvPSA2      CRLNKRQKIVLAQVFGASLPEQWRSKLA 308
SiPSA2B     SRLNKRQKVALAQVFGASLPDAVLLVFN 278
SiPSA2A     SRLNKRQKLALAQVFGASLPDVVLLVFS 322
SbPSA2      SRLNKRQKLVLAQVFGASLPDVVFLVSS 288
Consensus    r  kr   l q f     p
```
