# Supplementary material for: Characterization of the SWI/SNF complex and nucleosome organization in sorghum
Source: Front Plant Sci. 2024 Jun 26;15:1430467. doi: 10.3389/fpls.2024.1430467 (PMC11234113; doi:10.3389/fpls.2024.1430467)
Supplement: Supplementary Figure 1 — Domain architecture of the SWI/SNF complex subunits in six grass species. [file Image_1.pdf]

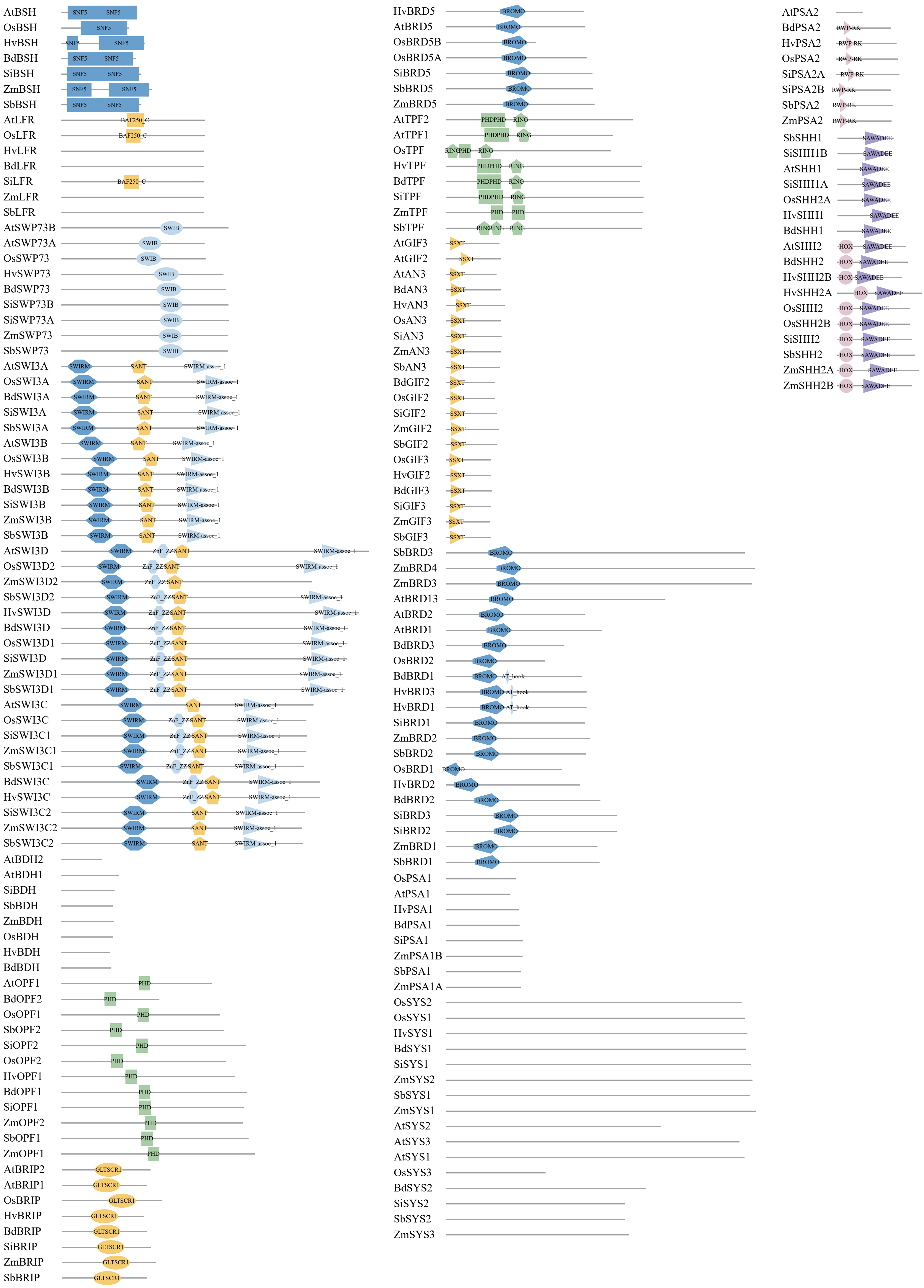

AtBSH SNF5 SNF5
OsBSH SNF5
HvBSH SNF5 SNF5
BdBSH SNF5 SNF5
SiBSH SNF5 SNF5
ZmBSH SNF5 SNF5
SbBSH SNF5 SNF5
AtLFR BAF250_C
OsLFR BAF250_C
HvLFR
BdLFR
SiLFR BAF250_C
ZmLFR
SbLFR
AtSWP73B SWIB
AtSWP73A SWIB
OsSWP73 SWIB
HvSWP73 SWIB
BdSWP73 SWIB
SiSWP73B SWIB
SiSWP73A SWIB
ZmSWP73 SWIB
SbSWP73 SWIB
AtSWI3A SWIRM SANT SWIRM-assoc_1
OsSWI3A SWIRM SANT SWIRM-assoc_1
BdSWI3A SWIRM SANT SWIRM-assoc_1
SiSWI3A SWIRM SANT SWIRM-assoc_1
SbSWI3A SWIRM SANT SWIRM-assoc_1
AtSWI3B SWIRM SANT SWIRM-assoc_1
OsSWI3B SWIRM SANT SWIRM-assoc_1
HvSWI3B SWIRM SANT SWIRM-assoc_1
BdSWI3B SWIRM SANT SWIRM-assoc_1
SiSWI3B SWIRM SANT SWIRM-assoc_1
ZmSWI3B SWIRM SANT SWIRM-assoc_1
SbSWI3B SWIRM SANT SWIRM-assoc_1
AtSWI3D SWIRM ZnF_ZZ SANT SWIRM-assoc_1
OsSWI3D2 SWIRM ZnF_ZZ SANT SWIRM-assoc_1
ZmSWI3D2 SWIRM ZnF_ZZ SANT
SbSWI3D2 SWIRM ZnF_ZZ SANT SWIRM-assoc_1
HvSWI3D SWIRM ZnF_ZZ SANT SWIRM-assoc_1
BdSWI3D SWIRM ZnF_ZZ SANT SWIRM-assoc_1
OsSWI3D1 SWIRM ZnF_ZZ SANT SWIRM-assoc_1
SiSWI3D SWIRM ZnF_ZZ SANT SWIRM-assoc_1
ZmSWI3D1 SWIRM ZnF_ZZ SANT SWIRM-assoc_1
SbSWI3D1 SWIRM ZnF_ZZ SANT SWIRM-assoc_1
AtSWI3C SWIRM SANT SWIRM-assoc_1
OsSWI3C SWIRM ZnF_ZZ SANT SWIRM-assoc_1
SiSWI3C1 SWIRM ZnF_ZZ SANT SWIRM-assoc_1
ZmSWI3C1 SWIRM ZnF_ZZ SANT SWIRM-assoc_1
SbSWI3C1 SWIRM ZnF_ZZ SANT SWIRM-assoc_1
BdSWI3C SWIRM ZnF_ZZ SANT SWIRM-assoc_1
HvSWI3C SWIRM ZnF_ZZ SANT SWIRM-assoc_1
SiSWI3C2 SWIRM SANT SWIRM-assoc_1
ZmSWI3C2 SWIRM SANT SWIRM-assoc_1
SbSWI3C2 SWIRM SANT SWIRM-assoc_1
AtBDH2
AtBDH1
SiBDH
SbBDH
ZmBDH
OsBDH
HvBDH
BdBDH
AtOPF1 PHD
BdOPF2 PHD
OsOPF1 PHD
SbOPF2 PHD
SiOPF2 PHD
OsOPF2 PHD
HvOPF1 PHD
BdOPF1 PHD
SiOPF1 PHD
ZmOPF2 PHD
SbOPF1 PHD
ZmOPF1 PHD
AtBRIP2 GLTSCR1
AtBRIP1 GLTSCR1
OsBRIP GLTSCR1
HvBRIP GLTSCR1
BdBRIP GLTSCR1
SiBRIP GLTSCR1
ZmBRIP GLTSCR1
SbBRIP GLTSCR1
HvBRD5 BROMO
AtBRD5 BROMO
OsBRD5B BROMO
OsBRD5A BROMO
SiBRD5 BROMO
SbBRD5 BROMO
ZmBRD5 BROMO
AtTPF2 PHDPHD RING
AtTPF1 PHDPHD RING
OsTPF RING PHD RING
HvTPF PHDPHD RING
BdTPF PHDPHD RING
SiTPF PHDPHD RING
ZmTPF PHD PHD
SbTPF RING RING RING
AtGIF3 SSXT
AtGIF2 SSXT
AtAN3 SSXT
BdAN3 SSXT
HvAN3 SSXT
OsAN3 SSXT
SiAN3 SSXT
ZmAN3 SSXT
SbAN3 SSXT
BdGIF2 SSXT
OsGIF2 SSXT
SiGIF2 SSXT
ZmGIF2 SSXT
SbGIF2 SSXT
OsGIF3 SSXT
HvGIF2 SSXT
BdGIF3 SSXT
SiGIF3 SSXT
ZmGIF3 SSXT
SbGIF3 SSXT
SbBRD3 BROMO
ZmBRD4 BROMO
ZmBRD3 BROMO
AtBRD13 BROMO
AtBRD2 BROMO
AtBRD1 BROMO
BdBRD3 BROMO
OsBRD2 BROMO
BdBRD1 BROMO AT_hook
HvBRD3 BROMO AT_hook
HvBRD1 BROMO AT_hook
SiBRD1 BROMO
ZmBRD2 BROMO
SbBRD2 BROMO
OsBRD1 BROMO
HvBRD2 BROMO
BdBRD2 BROMO
SiBRD3 BROMO
SiBRD2 BROMO
ZmBRD1 BROMO
SbBRD1 BROMO
OsPSA1
AtPSA1
HvPSA1
BdPSA1
SiPSA1
ZmPSA1B
SbPSA1
ZmPSA1A
OsSYS2
OsSYS1
HvSYS1
BdSYS1
SiSYS1
ZmSYS2
SbSYS1
ZmSYS1
AtSYS2
AtSYS3
AtSYS1
OsSYS3
BdSYS2
SiSYS2
SbSYS2
ZmSYS3
AtPSA2
BdPSA2 RWP-RK
HvPSA2 RWP-RK
OsPSA2 RWP-RK
SiPSA2A RWP-RK
SiPSA2B RWP-RK
SbPSA2 RWP-RK
ZmPSA2 RWP-RK
SbSHH1 SAWADEE
SiSHH1B SAWADEE
AtSHH1 SAWADEE
SiSHH1A SAWADEE
OsSHH2A SAWADEE
HvSHH1 SAWADEE
BdSHH1 SAWADEE
AtSHH2 HOX SAWADEE
BdSHH2 HOX SAWADEE
HvSHH2B HOX SAWADEE
HvSHH2A HOX SAWADEE
OsSHH2 HOX SAWADEE
OsSHH2B HOX SAWADEE
SiSHH2 HOX SAWADEE
SbSHH2 HOX SAWADEE
ZmSHH2A HOX SAWADEE
ZmSHH2B HOX SAWADEE
